# Binding between elongation factor 1A and the 3ʹ‐UTR of Chinese wheat mosaic virus is crucial for virus infection

**DOI:** 10.1111/mpp.13120

**Published:** 2021-08-17

**Authors:** Xuan Chen, Long He, Miaoze Xu, Jin Yang, Juan Li, Tianye Zhang, Qiansheng Liao, Hengmu Zhang, Jian Yang, Jianping Chen

**Affiliations:** ^1^ College of Plant Protection Northwest Agriculture and Forestry University Yangling China; ^2^ State Key Laboratory for Quality and Safety of Agro‐products Institute of Plant Virology of Ningbo University Ningbo China; ^3^ Institute of Virology and Biotechnology Zhejiang Academy of Agricultural Sciences Hangzhou China; ^4^ College of Life Science Zhejiang SCI‐Tech University Hangzhou China

**Keywords:** 3ʹ‐untranslated region, *Chinese wheat mosaic virus*, eukaryotic translation elongation factor eEF1A, stem‐loop, tRNA‐like structure, untranslated region

## Abstract

The Chinese wheat mosaic virus (CWMV) genome consists of two positive‐strand RNAs that are required for CWMV replication and translation. The eukaryotic translation elongation factor (eEF1A) is crucial for the elongation of protein translation in eukaryotes. Here, we show that silencing *eEF1A* expression in *Nicotiana benthamiana* plants by performing virus‐induced gene silencing can greatly reduce the accumulation of CWMV genomic RNAs, whereas overexpression of *eEF1A* in plants increases the accumulation of CWMV genomic RNAs. In vivo and in vitro assays showed that eEF1A does not interact with CWMV RNA‐dependent RNA polymerase. Electrophoretic mobility shift assays revealed that eEF1A can specifically bind to the 3ʹ‐untranslated region (UTR) of CWMV genomic RNAs. By performing mutational analyses, we determined that the conserved region in the 3ʹ‐UTR of CWMV genomic RNAs is necessary for CWMV replication and translation, and that the sixth stem‐loop (SL‐6) in the 3ʹ‐UTR of CWMV genomic RNAs plays a key role in CWMV infection. We conclude that eEF1A is an essential host factor for CWMV infection. This finding should help us to develop new strategies for managing CWMV infections in host plants.

## Graphical abstract

eEF1A facilitates CWMV infection in plants: it is involved in the replication and translation of CWMV via binding to the CWMV 3ʹ‐UTR. The sixth stem‐loop in the 3ʹ‐UTR is important for CWMV multiplication in infected cells.

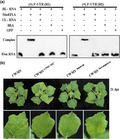

## 1 INTRODUCTION

Plant viruses have limited genome sizes, can encode only a few proteins, and thus must recruit host factor(s) to survive and replicate in infected cells (Ahlquist et al., 2003; Nagy, 2008; Noueiry & Ahlquist, 2003; Sanfaçon, 2015). Numerous host factors that influence plant virus infections in plants have been identified in recent years (Kang et al., 2005), but how these host factor(s) regulate virus replications in cells remains to be determined.

Eukaryotic elongation factors (eEFs), such as eEF1A and eEF1B, are key enzymes involved in protein elongation during translation in eukaryotes (Andersen & Nyborg, 2001). eEF1A is a highly abundant and conserved protein that delivers aminoacylated tRNA (aa‐tRNA) to the ribosome in a GTP‐dependent manner (Andersen et al., 2003). The noncanonical functions of eEF1A are vital for several cellular processes, including protein translation, cytoskeleton organization, nuclear export, and ubiquitin‐dependent protein degradation (Andersen et al., 2003; Vera et al., 2014). During protein synthesis, the ternary complex of eEF1A binds to and delivers aa‐tRNAs to the ribosome. When the aa‐tRNA anticodon in the ribosome matches the mRNA codon bound to the ribosome, GTP is hydrolysed to GDP and then combined with eEF1A. eEF1A is restored to active GTP under the action of the nucleotide exchange factor eEF1B (EF‐Ts). Finally, EF2 (EF‐G) assists the translocation of tRNA and mRNA through a codon on the ribosome. Moreover, eEF1A can also promote replication of certain RNA viruses through direct interaction with viral RNA‐dependent RNA polymerases (RdRp) or through interaction with viral genomic RNAs (Blackwell & Brinton, 1997; Davis et al., 2007). For example, eEF1A has been found to enhance the translation of turnip yellow mosaic virus (TYMV) RNA through binding to the 3ʹ‐untranslated region (3ʹ‐UTR) of viral genomic RNAs (Matsuda & Dreher, 2004). eEF1A can also bind to the upstream pseudoknot domain in the 3ʹ‐UTR of brome mosaic virus (BMV) RNAs and the 3ʹ‐UTR of tobacco mosaic virus (TMV) genomic RNA (Bastin & Hall, 1976; Joshi et al., 1986; Matsuda & Dreher, 2004; Zeenko et al., 2002). eEF1A can recruit TMV RdRp and the RdRp and VPg‐protease (VPg‐Pro) of turnip mosaic virus (TuMV) to form virus replication complexes (VRC) (Blumenthal et al., 1976; Thivierge et al., 2008; Yamaji et al., 2006). Furthermore, eEF1A can interact with the RdRp of tomato bushy stunt virus (TBSV) or West Nile virus (Davis et al., 2007; De Nova‐Ocampo et al., 2002; Rodnina & Wintermeyer, 2009; Sasvari et al., 2011).

Chinese wheat mosaic virus (CWMV) has two single‐strand positive‐sense genomic RNAs. *Chinese wheat mosaic virus* is a member of the genus *Furovirus*, family *Virgaviridae*. CWMV does not contain a poly(A) sequence at the RNA 3ʹ end of the genome (Adams et al., 2009). CWMV RNA1 consists of 7,147 nucleotides (nt) and encodes three proteins: a 153‐kDa replication‐associated protein, a 212‐kDa RdRp, and a 37‐kDa movement protein (MP). CWMV RNA2 comprises 3,564 nt and encodes four proteins: a 19‐kDa major capsid protein (CP), two minor CP‐related proteins (a 23‐kDa N‐CP and an 84‐kDa CP‐RT, produced through an initiation of translation at the noncanonical CUG start codon or through occasional read‐through of the UGA termination codon, respectively), and a 19‐kDa RNA silencing suppressor (Andika et al., 2013; Diao et al., 1999; Sun et al., 2013; Yang et al., 2001). Both the 5ʹ and 3ʹ termini of RNA1 and RNA2 contain UTRs. The 3ʹ‐UTR of RNA1 and RNA2 includes a highly conserved tRNA‐like structure, but the function of this structure is unclear. Full‐length CWMV infectious clones have recently been developed and used successfully to infect *Triticum aestivum* and *Nicotiana benthamiana* (Yang et al., 2016). Very few host factors have been identified to participate in CWMV infections or in other furovirus infections in plants.

Because eEF1A has been shown to regulate the replication of several RNA viruses, we decided to investigate the function of eEF1A in CWMV infection. We used in vivo assays to investigate whether eEF1A can positively influence CWMV infection in plants, as well as in vitro and reverse‐genetic assays to determine whether eEF1A can facilitate CWMV infection in plants via its binding to the 3ʹ‐UTR of CWMV genomic RNAs.

## 2 RESULTS

### 2.1 CWMV infection induces *eEF1A* expression in *T. aestivum* and *N. benthamiana*

**FIGURE 1:**
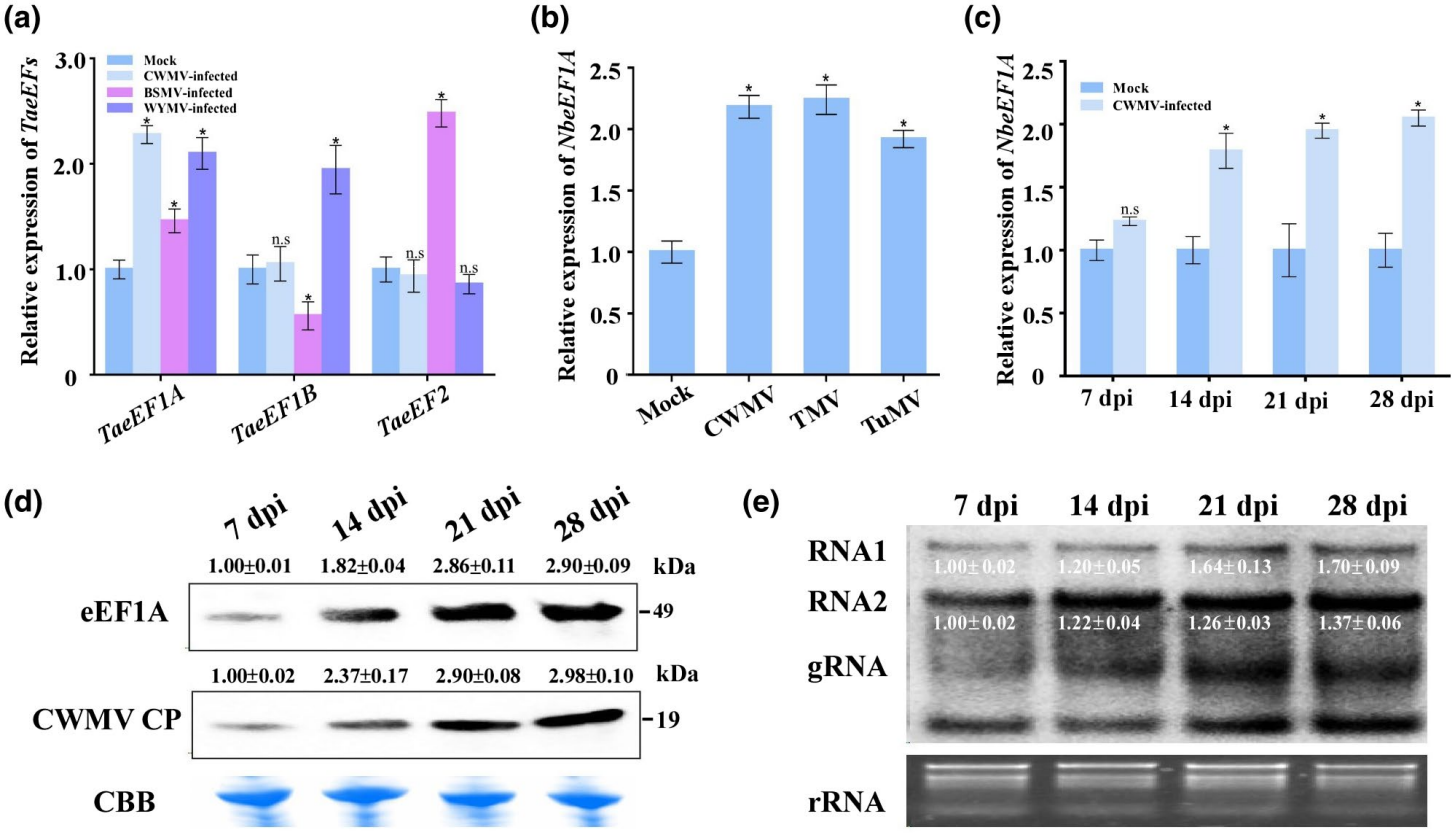
Relationship between the expression of eukaryotic translation elongation factors (*eEF*) and viral infection in plants. (a) Relative expression of *eEF*s in Chinese wheat mosaic virus (CWMV)‐, barley stripe mosaic virus (BSMV)‐, or wheat yellow mosaic virus (WYMV)‐infected or noninfected wheat (*Triticum aestivum*) plants at 14 days postinoculation (dpi). Wheat plants mock inoculated with inoculation buffer acted as controls. (b) Relative expression of *NbeEF1A* in CWMV‐, tobacco mosaic virus (TMV)‐, or turnip mosaic virus (TuMV)‐infected *Nicotiana benthamiana* plants. Plants infiltrated with the empty vector acted as a mock control. (c) Relative expression of *NbeEF1A* in CWMV‐infected *N*. *benthamiana* plants at 7 to 28 dpi. Plants mock infiltrated with the empty vector were used as a control. (d) Western blot assay for NbeEF1A and CWMV capsid protein (CP) expression at 7 to 28 dpi in CWMV‐infected *N. benthamiana* leaves using eEF1A‐ or a CP‐specific antibody, respectively. Coomassie Brilliant blue (CBB)‐stained RuBisCO gel was used to show protein loadings. (e) Northern blot assay for CWMV genomic RNAs (gRNAs) in CWMV‐infected *N. benthamiana* plants at 7 to 28 dpi. Ethidium bromide‐stained gel was used to visualize RNA loadings. The expression of *eEF* or CWMV *CP* genes was measured by quantitative reverse transcription PCR (RT‐qPCR) using gene‐specific primers. The relative expression level of these genes was calculated using the 2^−ΔΔ^ *^C^* ^t^ method. Data shown are the mean ± *SD* of three biological samples. Each biological sample had three technical replicates. Significant differences between treatments were determined using Student's *t* test (*p < 0.05; n.s., no significant difference). Viral RNAs and proteins were quantified using ImageJ software

To investigate the relationship between the expression of eEFs and CWMV infection in wheat plants, we studied three *T. aestivum* genes encoding eEFs: *TaeEF1A* (GenBank accession number AK334915), *TaeEF1B* (GenBank accession number AK332529), and *TaeEF2* (GenBank accession number AK452406). Barley stripe mosaic virus (BSMV, family *Hordevirus*) and wheat yellow mosaic virus (WYMV, family *Potyviridae*) are positive‐sense RNA viruses that can infect wheat. Quantitative reverse transcription PCR (RT‐qPCR) analyses were conducted using total RNA extracted from leaf samples harvested from CWMV‐, BSMV‐ or WYMV‐inoculated *T. aestivum* plants at 14 days postinoculation (dpi). Wheat plants mock inoculated with the empty vector were used as controls. Compared with the relative expression of *TaeEFs* in mock‐inoculated plants, the expression of *TaeEF1B* was downregulated by 0.6‐fold after BSMV infection and upregulated by 1.8‐fold after WYMV infection while no significant effects were observed after CWMV infection; the expression of *TaeEF2* was 2.3‐fold upregulated after BSMV infection while no significant effects were observed after CWMV or WYMV infection; and only the expression of *TaeEF1A* was significantly upregulated by approximately 1.4‐ to 2.3‐fold after CWMV, BSMV, or WYMV infection (Figure 1a). A homology‐based analysis revealed that the amino acid sequences of eEF1As from *Capsicum annuum*, *Solanum lycopersicum*, *Solanum pennellii*, *Solanum tuberosum*, *N. benthamiana*, *Ipomoea nil*, *Oryza sativa*, *Tarenaya hassleriana*, and *T. aestivum* share approximately 98% sequence identity (Figure S1). It has been reported that *NbeEF1A* is required both for TMV and TuMV infection (Thivierge et al., 2008; Zeenko et al., 2002). Thus, we decided to analyse the expression of *NbeEF1A* (XM_009784954) in *N. benthamiana* plants with or without viral infection by performing RT‐qPCR. The relative expression of *NbeEF1A* in CWMV‐, TMV‐, or TuMV‐infected *N. benthamiana* plants was significantly increased compared with expression in control plants that were mock infiltrated with the empty vector at 14 dpi (Figure 1b). Furthermore, we also analysed the relative expression of *NbeEF1A* in *N. benthamiana* plants at 7–28 days after CWMV infection by performing RT‐qPCR. The relative expression of *NbeEF1A* was approximately 2.2‐fold higher than that of mock‐treated plants 14 to 28 dpi after infection with CWMV (Figure 1c). Western blot and northern blot assays further showed that this correlated with increases in CWMV CP and CWMV genomic RNAs concentrations (Figure 1d,e). Thus, because *N. benthamiana* is an excellent experimental host plant for CWMV studies, and because an efficient and reliable agroinfiltration method has been developed for reverse‐genetic assays for CWMV infection (Yang et al., 2016, 2017), we decided to focus our research on the role of *NbeEF1A* in CWMV infection in subsequent experiments.

### 2.2 Silencing of *NbeEF1A* expression inhibits CWMV infection

**FIGURE 2:**
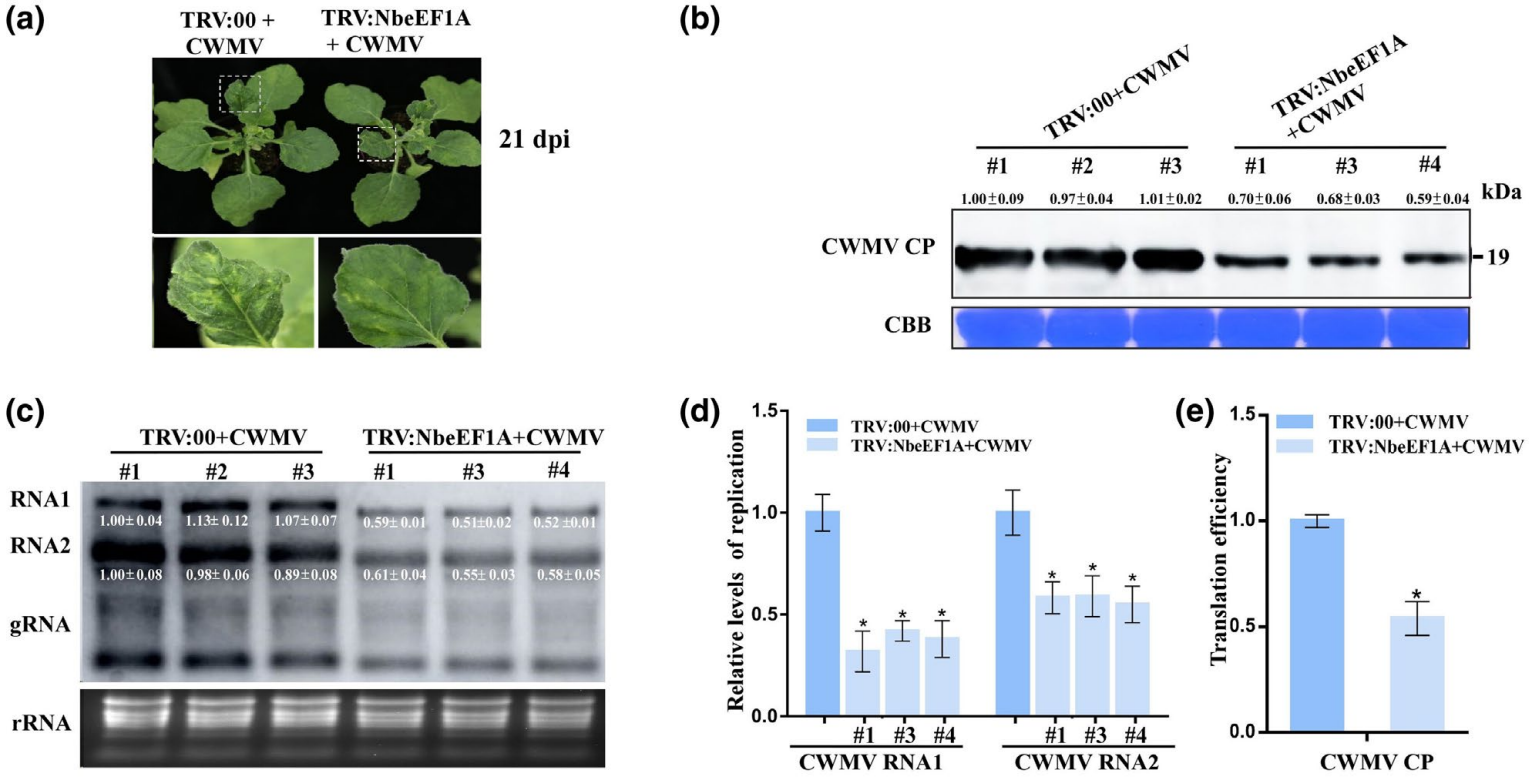
Silencing *NbeEF1A* in *Nicotiana benthamiana* attenuates CWMV infection. (a) Leaves of *N. benthamiana* plant #3 inoculated with TRV:00 and then CWMV (TRV:00 + CWMV) or TRV:NbeEF1A and then CWMV (TRV:NbeEF1A + CWMV) showing symptoms of disease at 21 days postinoculation (dpi). (b) Western blot assay for CWMV coat protein (CP) expression in TRV:NbeEF1A + CWMV‐inoculated leaves of assayed plants #1, #3, and #4 using a CP‐specific antibody at 21 dpi. Plants inoculated with TRV:00 + CWMV acted as controls. The Coomassie Brilliant blue (CBB)‐stained RuBisCO gel is used to show the protein loadings. Viral protein was quantified using ImageJ software. (c) Northern blot assay for CWMV RNA accumulation in TRV:NbeEF1A + CWMV‐inoculated leaves of assayed plants #1, #3, and #4. Plants inoculated with TRV:00 + CWMV acted as controls. Ethidium bromide‐stained gel was used to visualize RNA loadings. Viral RNAs were quantified using ImageJ software. (d) Relative transcript level of viral RNA in TRV:00 + CWMV‐inoculated or assayed *N. benthamiana* plants #1, #3, and #4 inoculated with TRV:NbeEF1A + CWMV. (e) Translation efficiency assay for CWMV CP in TRV:00 + CWMV‐inoculated plants and TRV:NbeEF1A + CWMV‐inoculated plants acted as control. Translation efficiency calculated as the relative expression in polysomal/total RNA fractions. Relative transcript levels are the mean ± *SD* of three biological samples; each biological sample had three technical replicates. Significant differences between treatments were determined using Student's *t* test (*p < 0.05)

A 400 nt sequence fragment of *NbeEF1A* was RT‐PCR amplified and cloned into a tobacco rattle virus (TRV)‐based vector to produce TRV:NbeEF1A. This vector was used to perform virus‐induced gene silencing (VIGS) in *N. benthamiana* plants via agroinfiltration. At 7 dpi, five assayed plants were sampled and analysed by performing RT‐qPCR. The results showed that *NbeEF1A* transcript levels in assayed plants #1, #3, and #4 were approximately 0.4‐ to 0.26‐fold lower than that in TRV:00‐inoculated control plants (Figure S2). To investigate the role of NbeEF1A in CWMV infection, we inoculated assayed plants #1, #3, and #4 with CWMV. Plants inoculated with TRV:00 and then CWMV (TRV:00 + CWMV) served as controls. At 21 dpi with CWMV, *NbeEF1A*‐silenced plants showed milder CWMV symptoms than TRV:00 + CWMV‐inoculated plants (Figure 2a). At 7 days after infection with CWMV, western blot and northern blot assays showed that significantly lower levels of CWMV CP and genomic RNAs were detected in CWMV‐inoculated #1, #3, and #4 plants than in TRV:00 + CWMV‐inoculated plants (Figure 2b,c). In addition, RT‐qPCR analyses also showed that the replication level of CWMV RNA1 and RNA2 in CWMV‐inoculated #1, #3, and #4 plants was significantly lower than that in leaves of TRV:00 + CWMV plants (Figure 2d). Due to the critical role of eEF1A in promoting mRNA translation, the effect of eEF1A on translation efficiency of CWMV genes was evaluated by RT‐qPCR analysis. The results showed that the translation efficiency of CWMV CP in CWMV‐inoculated *NbeEF1A*‐silenced plants was 0.54‐fold lower than that in TRV:00+CWMV plants (Figure 2e).

### 2.3 Overexpression of NbeEF1A in plants enhances CWMV accumulation

**FIGURE 3:**
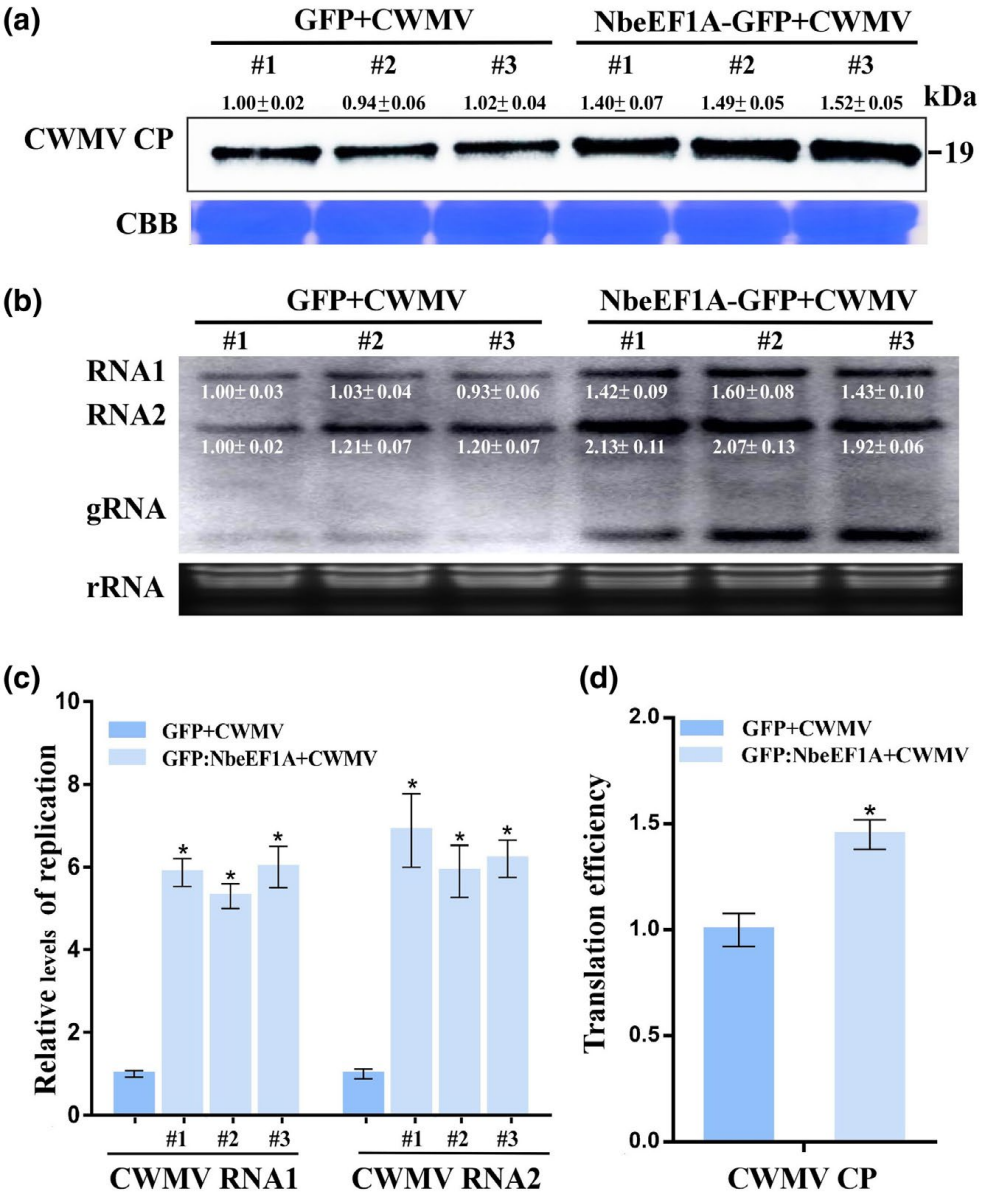
Transient overexpression of NbeEF1A in *Nicotiana benthamiana* plants enhances CWMV RNA accumulation. (a) Western blot assay for CWMV coat protein (CP) expression in plants coinoculated with CWMV and p35S:NbeEF1A‐GFP (NbeEF1A‐GFP + CWMV) through agroinfiltration using a CP‐specific antibody at 7 days postinoculation (dpi). Plants coinoculated with CWMV and p35S:GFP (GFP + CWMV) acted as controls. Proteins were resolved using sodium dodecyl sulphate polyacrylamide gel electrophoresis (SDS‐PAGE) stained with Coomassie Brilliant blue (CBB) to visualize protein loadings. Viral protein was quantified using ImageJ software. (b) Northern blot assay for CWMV RNA accumulation. Ethidium bromide‐stained gel was used to visualize RNA loadings. Three plants from the same treatment were used for the assay. Viral RNA was quantified using ImageJ software. (c) Relative transcript level of viral RNA in NbeEF1A‐GFP + CWMV‐inoculated plants and GFP + CWMV‐inoculated plants acted as controls. (d) Translation efficiency assay for CWMV CP in GFP + CWMV‐inoculated plants and NbeEF1A‐GFP + CWMV‐inoculated plants served as controls. Translation efficiency calculated as the relative expression in polysomal/total RNA fractions. Relative transcripts levels are the mean ± *SD* of three biological samples; each biological sample had three technical replicates. Significant differences between treatments were determined using Student's *t* test (*p < 0.05)

To further elucidate the role of NbeEF1A during CWMV infection, we constructed a p35S:NbeEF1A‐GFP expression vector to transiently overexpress NbeEF1A in *N. benthamiana* leaves. Leaves were coinfiltrated with *Agrobacterium* containing this expression vector and CWMV using agroinfiltration. Plants coinoculated with a green fluorescent protein (GFP) expression vector (p35S:GFP) and CWMV acted as controls. The expression of GFP and NbeEF1A‐GFP in infiltrated plants was confirmed at 3 dpi by performing a western blot assay using an anti‐GFP antibody (Figure S3). Total protein and RNA were extracted from infiltrated leaves at 7 dpi and accumulation of CWMV CP and genomic RNAs was analysed by performing western blot and northern blot assays, respectively. Accumulation of CWMV CP and genomic RNAs was higher in NbeEF1A‐GFP + CWMV‐inoculated plants than in control plants (Figure 3a,b). Moreover, RT‐qPCR analysis also showed that the replication level of CWMV RNA1 and RNA2 in the NbeEF1A‐GFP + CWMV‐inoculated plants was 5.7‐ to 6.3‐fold higher than that in leaves of plants coinoculated with GFP and CWMV (Figure 3c). As expected, the translation efficiency of CWMV CP in the NbeEF1A‐GFP + CWMV‐inoculated plants was 1.45‐fold higher than that in control plants (Figure 3d).

### 2.4 eEF1A binds to the 3ʹ‐UTR of CWMV genomic RNAs but not CWMV RdRp

**FIGURE 4:**
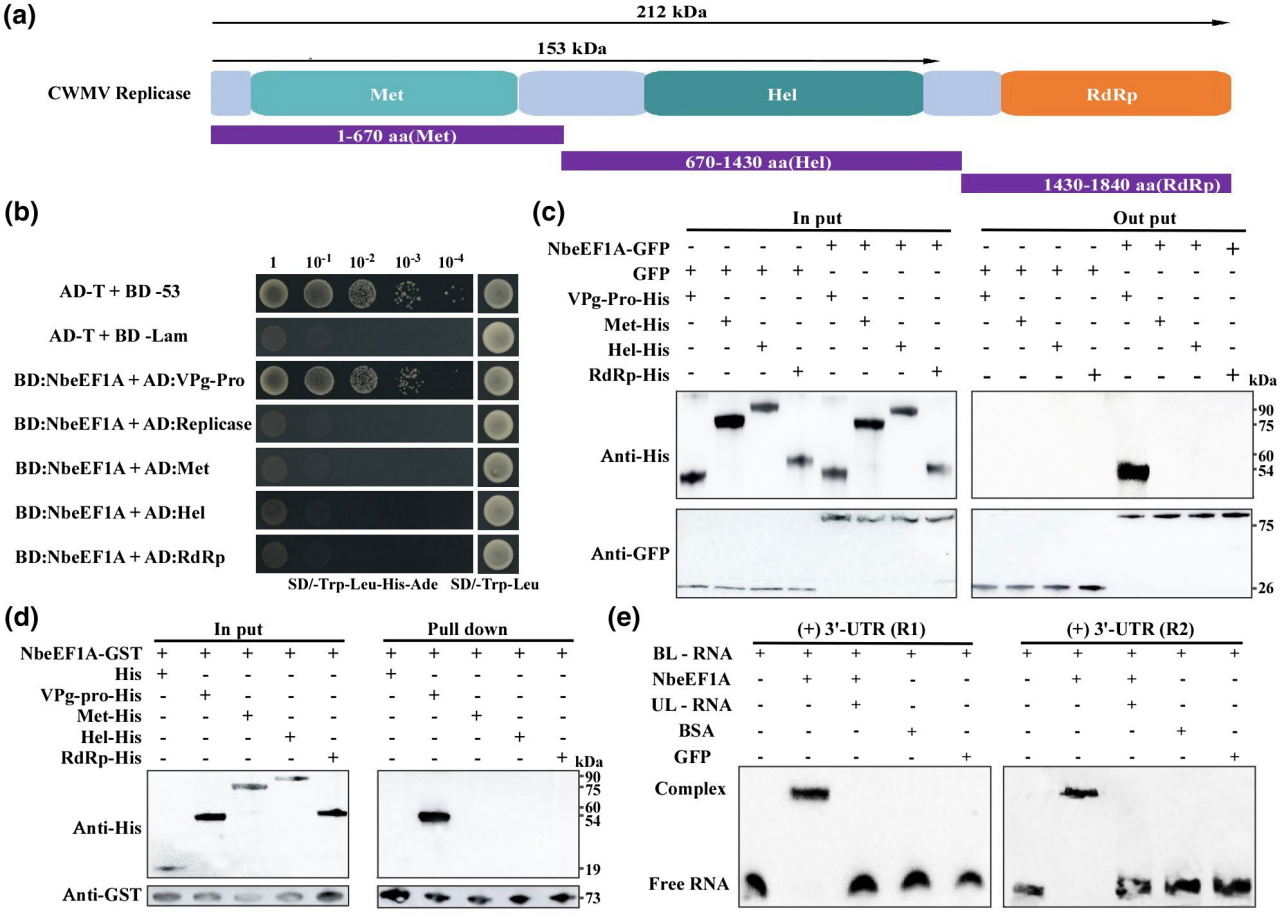
Analysis of the interaction between eEF1A and CWMV RdRp. (a) A schematic diagram showing CWMV replicase domains. The three segments (amino acid residues 1–670, 670–1430, and 1430–1840) represent the three conserved domains (Met, Hel, and RdRp) in CWMV replicase. (b) The right‐hand column shows that yeast cells coexpressing BD:NbeEF1A + AD:Replicase, BD:NbeEF1A + AD:Met, BD:NbeEF1A + AD:Hel, or BD:NbeEF1A + AD:RdRp grow well on SD/−Trp−Leu selective medium. The left‐hand column shows that none of the transformed yeast cells can grow on the SD/−Trp−Leu−His−Ade selective medium, indicating no positive interaction between NbeEF1A and the replicase domains. Yeast cells coexpressing AD‐T+BD‐53 or BD:NbeEF1A + AD:VPg‐Pro acted as positive controls. Yeast cells coexpressing AD‐T+BD‐Lam acted as a negative control. (c) Immunoprecipitation assay for binding of Met, Hel, and RdRp regions of CWMV to NbeEF1A, showing that binding failed in vivo. Immunoprecipitation was performed with anti‐GFP agarose beads and immunoblotting was carried out with anti‐His. GFP proteins acted as negative controls and TuMV VPg‐Pro‐His acted as positive controls. (d) Immunoprecipitation assay for binding of CWMV Met, Hel, and RdRp domains to NbeEF1A, showing that binding failed in vitro. A GST‐fused inactive NbeEF1A was first bound to glutathione–sepharose beads and incubated with Met‐His, Hel‐His, or RdRp‐His as indicated. His proteins acted as a negative control and TuMV VPg‐Pro acted as a positive control. (e) Electrophoretic mobility shift assay for NbeEF1A binding activity to (+) 3ʹ‐UTR of CWMV RNA1 (R1) or (+) 3ʹ‐UTR of CWMV RNA2 (R2). Each treatment has three components: a biotin‐labelled (BL) RNA probe, an unlabelled (UL) RNA probe (the UL‐RNA corresponds to the labelled RNA), and purified recombinant NbeEF1A or other protein. BL‐RNA + NbeEF1A was used to show the binding, UL‐RNA + BL‐RNA + NbeEF1A was used to show the competitive binding, and bovine serum albumen (BSA) and green fluorescent protein (GFP) acted as negative controls

**FIGURE 5:**
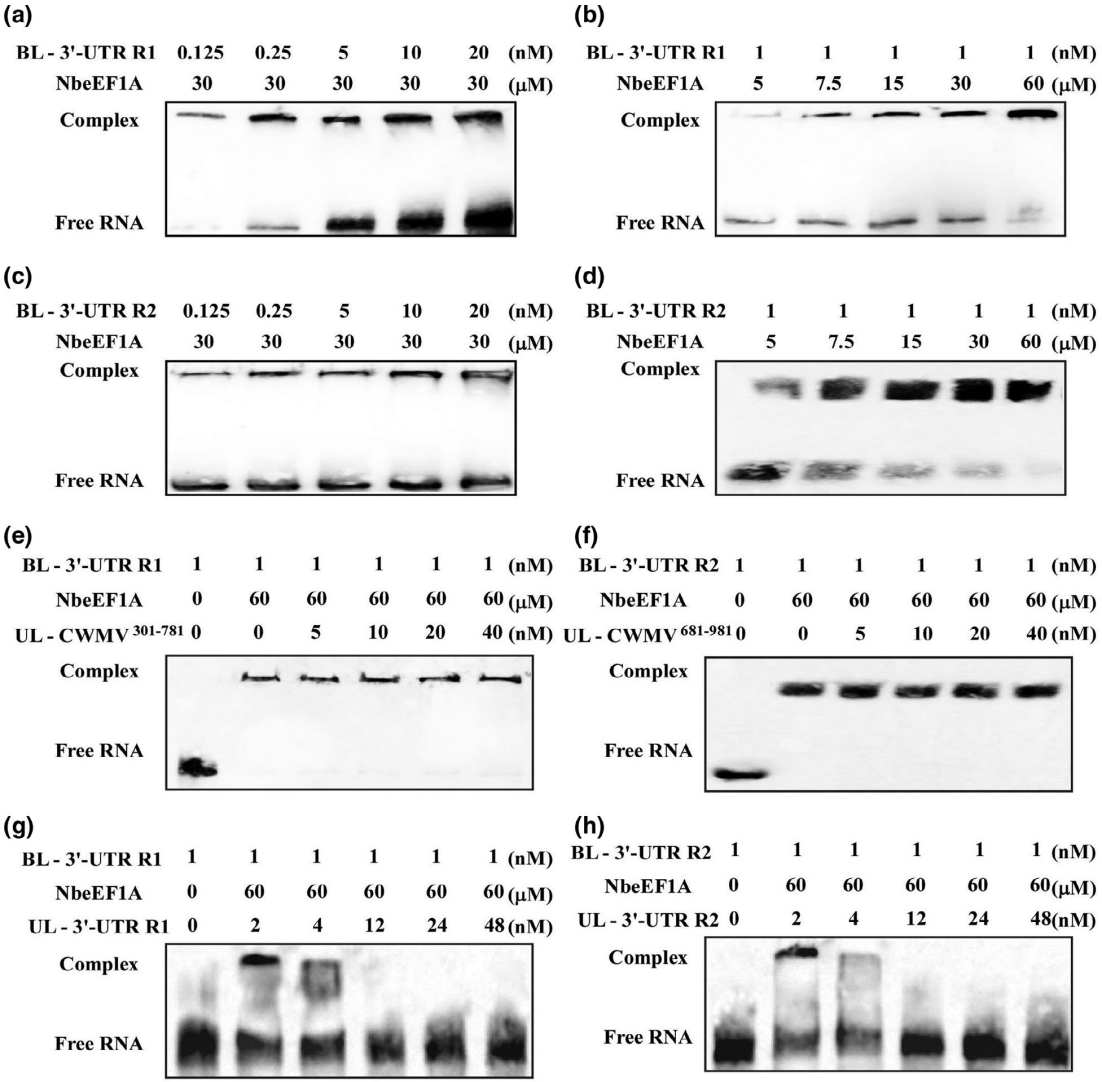
Electrophoretic mobility shift assays for competitive binding between NbeEF1A and (+) 3ʹ‐UTR (R1), (+) 3ʹ‐UTR (R2), UL‐CWMV^301–781^, or UL‐CWMV^681–981^. (a) Protein concentration‐dependent binding activity of NbeEF1A for CWMV (+) 3ʹ‐UTR of RNA1 (R1). The amount added to each component is indicated at the top. (b) Biotin‐labelled (BL)‐RNA concentration‐dependent binding activity of NbeEF1A for CWMV (+) 3ʹ‐UTR (R1). The amount added to each component is indicated at the top. (c) Protein concentration‐dependent binding activity of NbeEF1A for CWMV (+) 3ʹ‐UTR of RNA2 (R2). The amount added to each component is indicated at the top. (d) BL‐RNA concentration‐dependent binding activity of NbeEF1A for CWMV (+) 3ʹ‐UTR (R2). The amount added to each component is indicated at the top. (e) Assay for competitive binding of BL‐3ʹ‐UTR (R1) + NbeEF1A and unlabelled (UL)‐CWMV^301–781^ of RNA1. The amount added to each component is indicated at the top. (f) Assay for competitive binding of BL‐3ʹ‐UTR (R1) + NbeEF1A and UL‐CWMV^681–981^ of RNA2. The amount added to each component is indicated at the top. (g) Titration of a UL‐3ʹ‐UTR (R1) with NbeEF1A + BL‐3ʹ‐UTR (R1). The amount added to each component is indicated at the top. (h) Titration of a UL‐3ʹ‐UTR (R2) with NbeEF1A + BL‐3ʹ‐UTR (R2). The amount added to each component is indicated at the top

The successful infection of a plant by an RNA virus and disease symptom development are dependent on complicated molecular interactions between viral and host factors. Numerous reports have indicated that eEFs can interact with viral RdRps and/or viral genomic RNAs (Dufresne et al., 2008; Li et al., 2010, 2015; Matsuda & Dreher, 2004; Thivierge et al., 2008; Yamaji et al., 2006). To determine whether CWMV replication‐associated proteins can interact with NbeEF1A, we performed yeast two‐hybrid assays, coimmunoprecipitation (Co‐IP) assays, and pull‐down assays. Because the full‐length replication‐associated protein has a large molecular weight, the CWMV replication‐associated protein (Rep) was divided into three fragments according to their functional domains: amino acid positions 1–670 referred to as Rep^1–670^ (methyltransferase, Met), amino acid positions 670–1430 referred to as Rep^670–1430^ (helicase, Hel), and amino acid positions 1430–1840 referred to as Rep^1430–1840^ (RdRp) (Figure 4a). As shown in Figure 4b, all the yeasts harbouring a plasmid expressing BD‐eEF1A together with a plasmid expressing AD‐replicase, ‐Met, ‐Hel, or ‐RdRp, did not show any growth on the selection medium compared with the positive control. Additionally, the interaction between Met‐, Hel‐, or RdRp‐His and NbeEF1A‐GFP in planta was confirmed by a Co‐IP assay after transiently coexpressing Met‐, Hel‐, or RdRp‐His, and NbeEF1A‐GFP in *N. benthamiana* leaves, respectively. The interaction of Met‐, Hel‐, or RdRp‐His with NbeEF1A‐GST was also observed by pull‐down assays. The VPg‐Pro protein of TuMV was used as a positive control in these assays. Our results showed that although the VPg‐Pro protein interacted with NbeEF1A, none of the CWMV replication‐associated protein domains interacted with NbeEF1A (Figure 4b–d). Further analyses also showed that these three domains did not interact with TaeEF1A in yeast cells (Figure S4). eEF1A has been reported to interact with viral RNAs to ensure RNA virus replication in cells (Blackwell & Brinton, 1997; Davis et al., 2007). Here, we decided to explore whether NbeEF1A can bind to CWMV genomic RNAs by performing electrophoretic mobility shift assays (EMSA). We prepared in vitro transcripts of the CWMV (+/−) 3ʹ‐UTR and (+/−) 5ʹ‐UTR of RNA1 and RNA2, which were biotin labelled (BL) at their 3ʹ ends (+/−). In addition, CWMV^301–781^ of RNA1 and (+/−) CWMV^681–981^ of RNA2 (regions covering nucleotides 301–781 or 681–981, respectively, in the CWMV coding region) were randomly selected and BL at their 3ʹ ends. Binding of eEF1A to the 3ʹ‐UTRs of RNA1 and RNA2 was analysed by EMSA. Shift of the 3ʹ‐UTR bands was observed on addition of eEF1A and disappeared after adding competitive cold unlabelled RNA (UL‐RNA) (Figure 4e), but not in other treated reactions (Figure S5a,b). We examined the effects of different concentrations of BL‐3ʹ‐UTR transcripts on the formation of the 3ʹ‐UTR RNA1 (R1)/NbeEF1A and 3′‐UTR RNA2 (R2)/NbeEF1A complexes. The results showed an increase in the retarded band when 3′‐UTR RNA or NbeEF1A concentrations were increased from 0.125 to 20 nM or 5 to 60 µM, respectively, supporting this interaction (Figure 5a–d). The assays also showed no effect on the formation of the 3ʹ‐UTR RNA/NbeEF1A complex when unlabelled CWMV^301–781^ RNA1 (UL‐CWMV^301‐781^) or CWMV^681–981^ RNA2 (UL‐CWMV^681‐981^) concentrations were increased from 0 to 40 nM (Figure 5e,f). Moreover, the binding activity between NbeEF1A and 3ʹ‐UTR RNA was weakened following the addition of excess UL‐3ʹ‐UTR RNA from 0 to 48 nM (Figure 5g,h). These findings suggest that eEF1A can bind to the 3ʹ‐UTR of CWMV genomic RNAs but cannot interact with CWMV RdRp.

### 2.5 A conserved region in the 3ʹ‐UTR is crucial for CWMV infection

**FIGURE 6:**
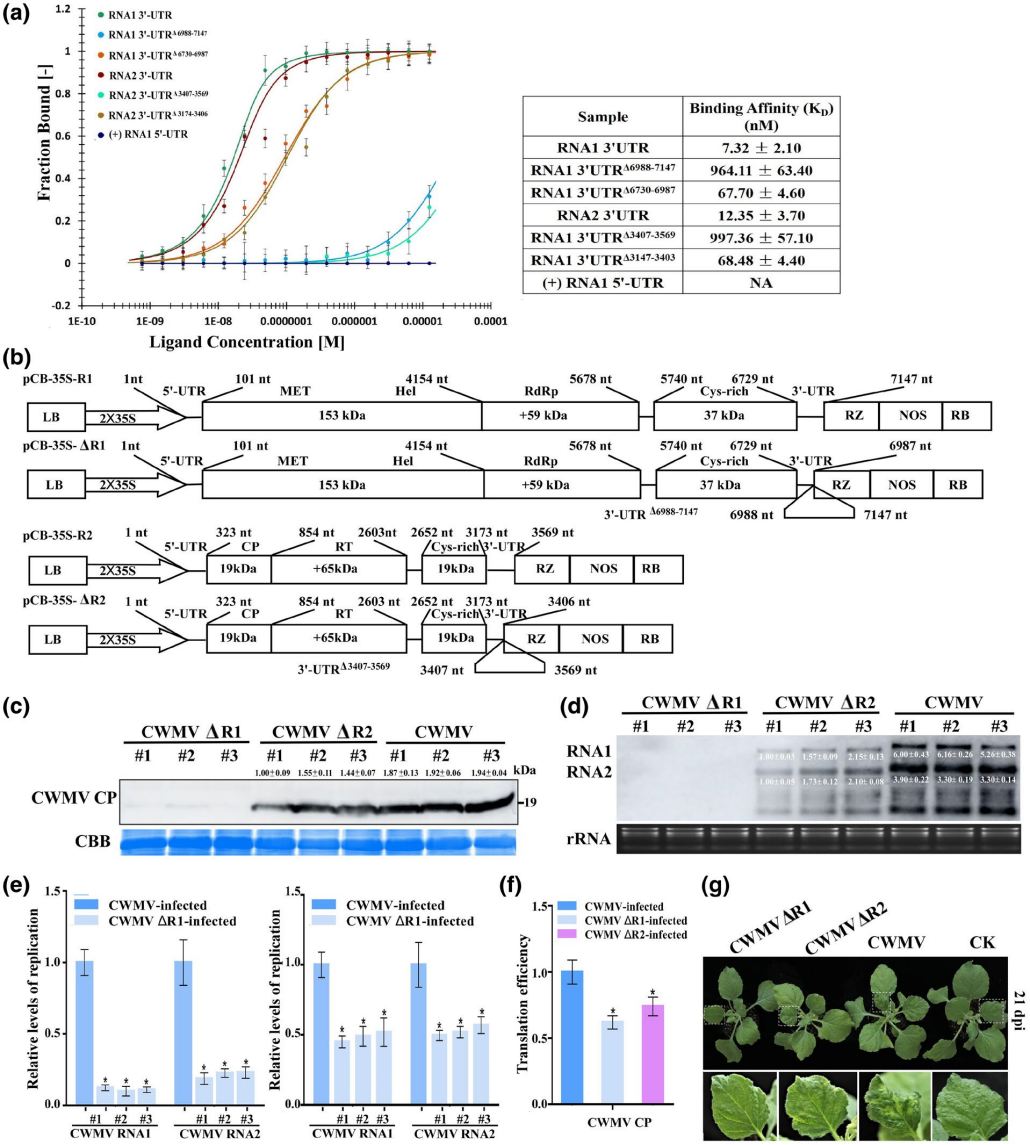
The 3ʹ‐UTR of CWMV RNA is necessary for viral infection. (a) A microscale thermophoresis assay of NbeEF1A binding affinity for RNA1 3ʹ‐UTR, RNA1 3ʹ‐UTR^Δ6988–7147^, RNA1 3ʹ‐UTR^Δ6730–6987^, RNA2 3ʹ‐UTR, RNA2 3ʹ‐UTR^Δ3407–3569^, or RNA2 3ʹ‐UTR^Δ3174–3406^ transcripts. RNA1 5ʹ‐UTR transcripts acted as a negative control. This experiment was performed three times and similar results were obtained. Bars represent standard errors. NA, no affinity. (b) Schematic diagrams showing the difference between the wild type (WT) and the deletion mutants CWMV ΔRNA1 and CWMV ΔRNA2. (c) Western blot assay for CWMV coat protein (CP) accumulation in WT CWMV‐, CWMV ΔR1‐, or CWMV ΔR2‐inoculated *Nicotiana benthamiana* plants. Coomassie Brilliant blue (CBB) was used to visualize protein loadings. Three plants from the same treatment were used. The relative intensity of the blot signal quantified by ImageJ is shown above the lane. (d) Northern blot assay for CWMV RNA accumulation in WT CWMV‐, CWMV ΔR1‐, or CWMV ΔR2‐inoculated *N. benthamiana* plants. Ethidium bromide‐stained gel was used to visualize RNA loadings. Three plants from the same experiment were used. The relative intensity of the blot signal quantified by ImageJ is shown in the image. (e) Relative levels of CWMV RNA1 and RNA2 replication assessed by quantitative reverse transcription PCR in WT CWMV‐, CWMV ΔR1‐, or CWMV ΔR2‐inoculated *N. benthamiana* leaves. (f) Translation efficiency assay for CWMV CP in CWMV ΔR1‐ or CWMV ΔR2‐inoculated *N. benthamiana* plants and WT CWMV‐inoculated plants served as controls. Translation efficiency calculated as the relative expression in polysomal/total RNA fractions. (g) Symptoms in WT CWMV‐, CWMV ΔR1‐, or CWMV ΔR2‐inoculated *N. benthamiana* plants at 21 days postinoculation (dpi) with CWMV. Data presented are the mean ± *SD* from three biological samples per treatment; each biological sample had three technical replicates. Significant differences between treatments were determined using Student's *t* test (*p < 0.05)

The sequences of the 3ʹ‐UTRs of CWMV RNA1 and RNA2 have an identity of approximately 57%, but interestingly a region of about 160 nucleotides of RNA1 and RNA2 (nucleotide positions 6988–7147 and 3407–3569) shows higher similarity (Figure S6a). Secondary structure predictions using Mfold software (http://unafold.rna.albany.edu/?q=mfold) indicated that there is a tRNA‐like structure (TLS) at the conserved region of the 3ʹ‐UTR of CWMV RNA1 or RNA2 (Figure S6b). We conducted a microscale thermophoresis (MST) of NbeEF1A and the RNA1 3ʹ‐UTR, RNA2 3ʹ‐UTR, RNA1 3ʹ‐UTR^Δ6730–6987^, RNA2 3ʹ‐UTR^Δ3174–3406^, RNA1 3ʹ‐UTR^Δ6988–7147^, or RNA2 3ʹ‐UTR^Δ3407–356^, respectively. The binding equilibrium dissociation constant (*K*_D_) values between NbeEF1A and the RNA1 3ʹ‐UTR, RNA2 3ʹ‐UTR, RNA1 3ʹ‐UTR^Δ6730–6987^, RNA2 3ʹ‐UTR^Δ3174–3406^, RNA1 3ʹ‐UTR^Δ6988–7147^, or RNA2 3ʹ‐UTR^Δ3407–3569^ were 7.32 ± 2.1 nM, 12.53 ± 3.7 nM, 67.70 ± 4.6 nM, 68.48 ± 4.4 nM, 964.11 ± 63.4 nM, or 997.36 ± 57.1 nM, respectively (Figure 6a). The CWMV^301–781^ of RNA1 was used as a control. Our analyses demonstrated that the conserved TLS in the 3ʹ‐UTR is a key domain response for NbeEF1A binding CWMV genomic RNAs. To elucidate the function of the TLS during CWMV infection, we generated a mutant CWMV ΔR1 with the TLS deleted in its 3ʹ‐UTR (RNA1 3ʹ‐UTR^Δ6988–7147^) and a ΔR2 with the TLS deleted in its 3ʹ‐UTR (RNA2 3ʹ‐UTR^Δ3407–3569^) (Figure 6b). Next, CWMV ΔR1 and ΔR2 were coinoculated with the wildtype (WT) CWMV RNA2 or RNA1, respectively, into *N. benthamiana* plants using agroinfiltration. WT CWMV RNA1 and RNA2 coinoculated plants served as controls. At 7 dpi, CP and viral RNA accumulations were much lower in CWMV ΔR2‐inoculated plants than those in WT CWMV‐inoculated plants and were hardly detected in CWMV ΔR1‐inoculated plants (Figure 6c,d). RT‐qPCR analyses also showed that CWMV RNA1 and RNA2 replication levels were 0.20‐ to 0.31‐fold lower in CWMV ΔR1‐inoculated plants and 0.50‐ to 0.58‐fold lower in CWMV ΔR2‐inoculated plants than in WT CWMV‐inoculated plants (Figure 6e). Consistent with these findings, the translation efficiency of CWMV CP was 0.62‐ to 0.74‐fold lower in CWMV ΔR1‐inoculated plants or CWMV ΔR2‐inoculated plants, respectively, than in WT CWMV‐inoculated plants (Figure 6f). By 21 dpi, CWMV ΔR2‐inoculated *N*. *benthamiana* plants showed much milder disease symptoms than WT CWMV‐inoculated plants, whereas CWMV ΔR1‐inoculated plants showed mild disease symptoms that were similar to those of WT plants (Figure 6g). Taken together, our analyses demonstrated that the conserved TLS in the 3ʹ‐UTR is a key domain for NbeEF1A binding CWMV genomic RNAs, which is crucial for CWMV infection.

### 2.6 Role of stem‐loop structures in CWMV RNA2 3ʹ‐UTR in the interaction between NbeEF1A and 3ʹ‐UTR

**FIGURE 7:**
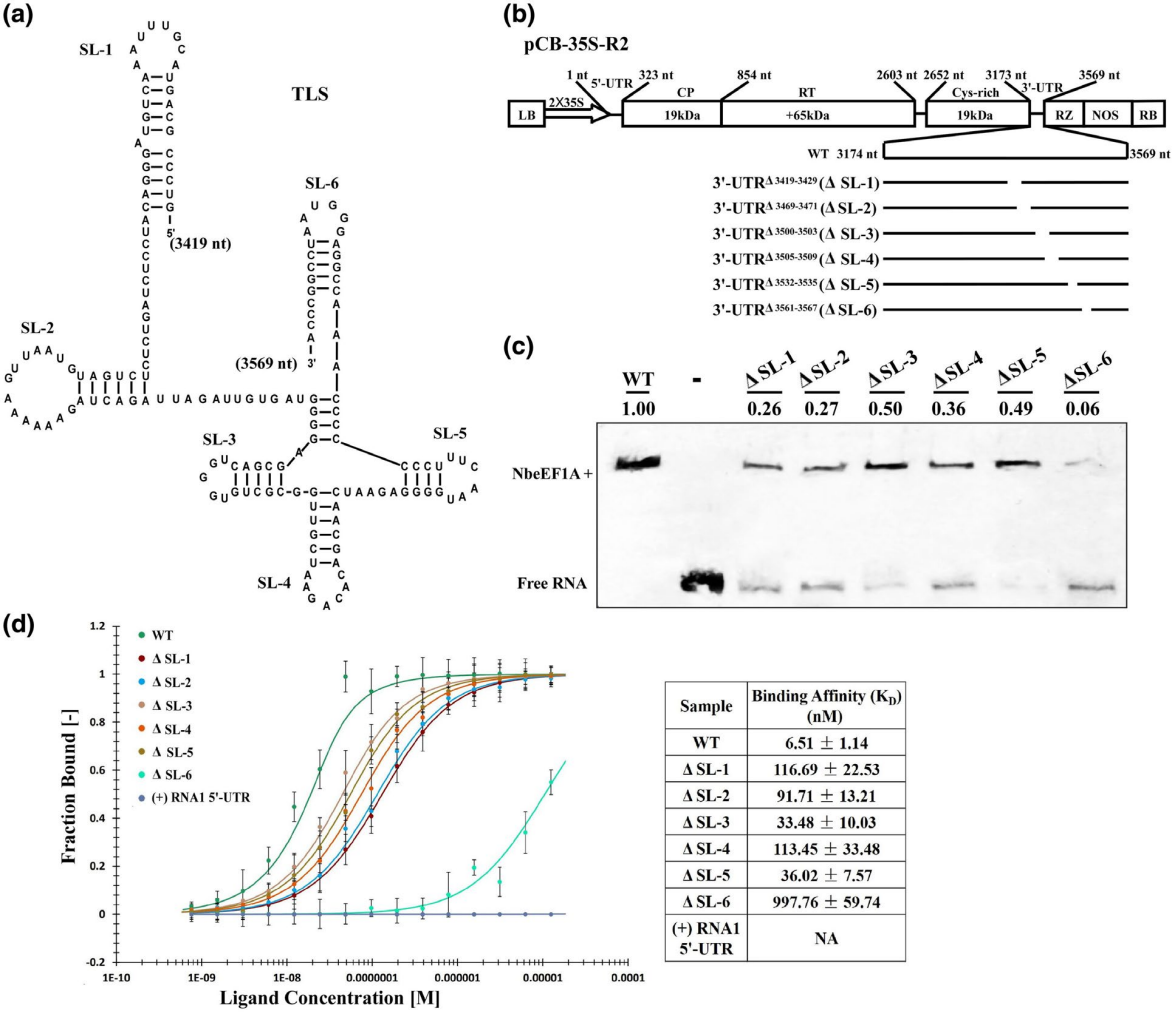
Effect of stem‐loop (SL)‐like structures in the CWMV RNA2 3ʹ‐UTR on binding with NbeEF1A. (a) Prediction of six SL‐like structures (SL‐1 to SL‐6) in the CWMV RNA2 3ʹ‐UTR (nucleotide positions 3419–3569). (b) A schematic diagram of the wildtype (WT) CWMV RNA2 construct and the six SL deletion mutants. (c) Electrophoretic mobility shift assays of NbeEF1A binding activity for the six mutant RNA transcripts (Δ1–Δ6). The reaction with WT CWMV RNA2 3ʹ‐UTR transcripts acted as a control. (d) Microscale thermophoresis assay for NbeEF1A binding affinity for the WT 3ʹ‐UTR RNA and that of six mutant RNA transcripts. This experiment was performed three times and similar results were obtained. Bars represent standard errors

Given that the TLS is highly conserved in CWMV RNA1 and RNA2 and that the deletion of the TLS from CWMV RNA1 3ʹ‐UTR affected CWMV infection, we decided to further investigate the TLS of CWMV RNA2. Predictions using CWMV RNA2 3ʹ‐UTR (3419–3569 nucleotides) revealed six stem‐loop (SL)‐like structures that form the TLS (Figure 7a). To determine which SL structure affects the binding affinity of CWMV 3ʹ‐UTR for NbeEF1A, we prepared six CWMV RNA2 3ʹ‐UTR deletion mutants (i.e., ΔSL‐1 for 3ʹ‐UTR^Δ3419–3429^, ΔSL‐2 for 3ʹ‐UTR^Δ3469–3471^, ΔSL‐3 for 3ʹ‐UTR^Δ3500–3503^, ΔSL‐4 for 3ʹ‐UTR^Δ3505–3509^, ΔSL‐5 for 3ʹ‐UTR^Δ3532–3535^, and ΔSL‐6 for 3ʹ‐UTR^Δ3561–3565^) (Figure 7b). We analysed the binding activity between these mutants and NbeEF1A, which indicated that all of these mutants did affect the binding activity. Compared with the WT 3ʹ‐UTR, the shifts of the ΔSL‐1, ΔSL‐2, ΔSL‐3, ΔSL‐4, and ΔSL‐5 bands were significantly weakened, and the most reduced ΔSL‐6 was only 6% of the WT 3ʹ‐UTR (Figure 7c). To further measure the binding affinity between NbeEF1A and 3ʹ‐UTR, we conducted an MST assay using the same concentration of NbeEF1A and the indicated amounts of these six mutant transcripts. The assays revealed a *K*_D_ between NbeEF1A and the WT 3ʹ‐UTR, ΔSL‐1, ΔSL‐2, ΔSL‐3, ΔSL‐4, or ΔSL‐5 of 6.51 ± 1.14 nM, 116.69 ± 22.53 nM, 91.71 ± 13.21 nM, 33.48 ± 10.03 nM, 113.45 ± 33.48 nM, and 36.02 ± 7.57 nM, respectively (Figure 7d), indicating that these mutant 3ʹ‐UTRs all had lower binding capacities for NbeEF1A than that of the WT 3ʹ‐UTR. In addition, the mutant ΔSL‐6 had the highest *K*_D_ value (997.76 ± 59.74 nM), which supports the above finding that the TLS structure is required for the interaction between NbeEF1A and 3ʹ‐UTR, and that SL‐6 may be the key region in the TLS for this interaction.

### 2.7 SL‐6 in 3ʹ‐UTR is crucial for CWMV accumulation

**FIGURE 8:**
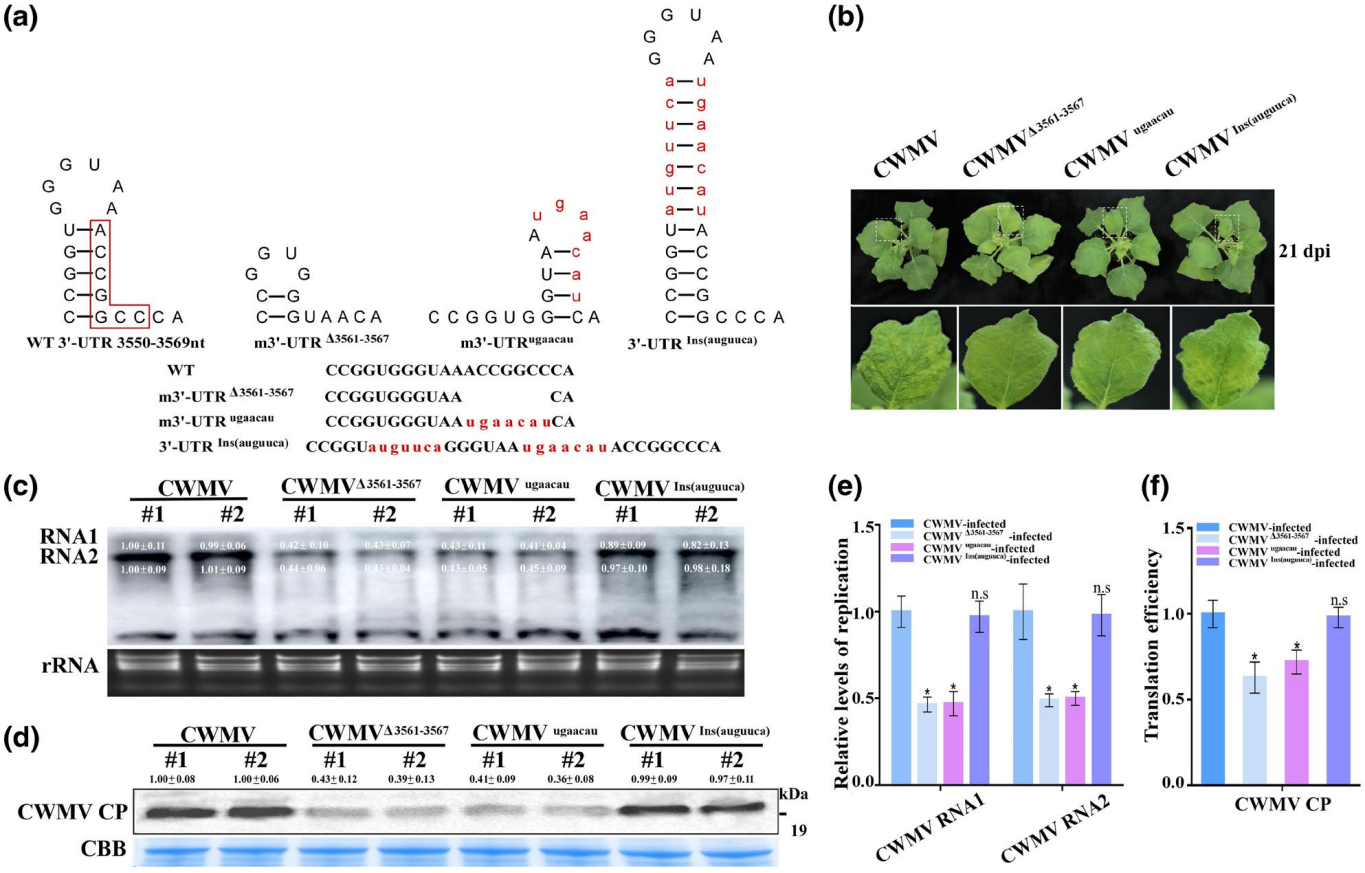
Effect of the sixth stem‐loop (SL) structure in CWMV RNA2 3ʹ‐UTR on CWMV infection. (a) Predicted structures of SL‐6 in the wildtype (WT) CWMV RNA2 3ʹ‐UTR, the m3ʹ‐UTR^ugaacau^, the m3ʹ‐UTR^Δ3561–3567^, or 3ʹ‐UTR^Ins(auguuca)^. The red box indicates the region where the mutation is made. Red letters indicate the site where the mutation is made. (b) Symptoms in WT CWMV‐, CWMV^Δ3561–3567^‐, CWMV^ugaacau^‐, or CWMV^Ins(auguuca)^ ‐inoculated *Nicotiana benthamiana* plants at 21 days postinoculation (dpi) with CWMV. (c) Northern blot assay for CWMV RNA accumulation in WT CWMV‐, CWMV^Δ3561–3567^‐, CWMV^ugaacau^‐, or CWMV^Ins(auguuca)^ ‐inoculated *N. benthamiana* plants at 21 dpi. Ethidium bromide‐stained gel was used to visualize RNA loadings. Two plants from the same treatment were used for the assay. Viral RNA was quantified using ImageJ software. (d) Western blot assay for CWMV coat protein (CP) accumulation in WT CWMV‐, CWMV^Δ3561–3567^‐, CWMV^ugaacau^‐, or CWMV^Ins(auguuca)^‐inoculated *N. benthamiana* plants. Coomassie Brilliant blue (CBB) was used to visualize the protein loadings. Two plants from the same treatment were used. The relative intensity of the blot signal quantified by ImageJ is shown above the lane. (e) Relative levels of CWMV RNA1 and RNA2 replication quantification by quantitative reverse transcription PCR in WT CWMV‐, CWMV^Δ3561–3567^‐, CWMV^ugaacau^‐, or CWMV^Ins(auguuca)^ ‐inoculated *N. benthamiana* leaves. (f) Translation efficiency assay for CWMV CP in CWMV^Δ3561–3567^‐, CWMV^ugaacau^‐, or CWMV^Ins(auguuca)^ ‐inoculated *N. benthamiana* plants and WT CWMV‐inoculated plants served as controls. Translation efficiency calculated as the relative expression in polysomal/total RNA fractions. Data presented are the mean ± *SD* of three biological samples per treatment. Each biological sample had three technical replicates. Significant differences between treatments were determined using Student's *t* test (*p < 0.05)

To investigate the function of SL‐6 in eEF1A‐regulated CWMV accumulation, we changed the ACCGGCC of SL‐6 to ugaacau for producing the mutant m3ʹ‐UTR^ugaacau^, and then complementary mutations in the opposite strand to produce the mutant 3ʹ‐UTR^Ins(auguuca)^ or by deleting nucleotide positions 3561–3567 of the RNA2 3ʹ‐UTR to produce the mutant m3ʹ‐UTR^Δ3561–3567^. As shown in Figure 8a, the stem structure was changed in the mutant m3ʹ‐UTR^ugaacau^ and m3ʹ‐UTR^Δ3561–3567^, while it was restored in the complementary mutant 3ʹ‐UTR^Ins(auguuca)^. *Agrobacterium* harbouring the CWMV RNA1 and RNA2 clones (CWMV) or the CWMV RNA1 and the m3ʹ‐UTR^ugaacau^ clones (CWMV^ugaacau^) or the CWMV RNA1 and the m3ʹ‐UTR^Δ3561–3567^ clones (CWMV^Δ3561–3567^) or the CWMV RNA1 and the 3ʹ‐UTR^Ins(auguuca)^ clones (CWMV^Ins(auguuca)^) were inoculated into *N. benthamiana* plants using agroinfiltration. By 21 dpi, both CWMV^ugaacau^‐ and CWMV^Δ3561–3567^‐inoculated *N. benthamiana* plants showed much milder disease symptoms than CWMV‐inoculated plants (Figure 8b) and accumulated significantly lower levels of viral RNAs and CP (Figure 8c,d). Interestingly, CWMV^Ins(auguuca)^‐inoculated *N. benthamiana* plants showed similar disease symptoms at 21 dpi (Figure 8b). Western blot and northern blot assays showed greater CWMV RNA and CP accumulations in WT CWMV‐ and CWMV^Ins(auguuca)^‐inoculated *N. benthamiana* plants at 21 dpi than in other plants, which was consistent with these observations (Figure 8c,d). In addition, RT‐qPCR also showed that the replication level of CWMV RNA1 and RNA2 in the CWMV^ugaacau^‐ or CWMV^Δ3561–3567^‐inoculated plants was 0.50‐ to 0.51‐fold lower, respectively, than that in CWMV‐inoculated plants (Figure 8e). By contrast, replication levels of CWMV RNA1 and RNA2 in CWMV^Ins(auguuca)^‐inoculated plants were higher than those in CWMV^ugaacau^‐ or CWMV^Δ3561–3567^‐inoculated plants and accumulation levels were similar to that in CWMV‐inoculated plants (Figure 8e). The translation efficiencies of CWMV CP in CWMV^ugaacau^‐ or CWMV^Δ3561–3567^‐inoculated plants were 0.72‐ to 0.63‐fold lower while that in CWMV^Ins(auguuca)^‐inoculated plants was similar than that in WT CWMV‐inoculated plants (Figure 8f). Thus, we conclude that the SL‐6 in the 3ʹ‐UTR is important for CWMV multiplication in infected cells.

## 3 DISCUSSION

Because viruses have limited coding capacities, they must rely on host factor(s) to complete their infection cycles in cells (Nagy & Pogany, 2011). One of the best‐studied host factors is eEF1A. Many studies on the roles of eEF1A during virus infections of plants have demonstrated that eEF1A can positively regulate the multiplication of viruses such as TMV, BSMV, TuMV, and tomato spotted wilt virus (Komoda et al., 2014; Li et al., 2009, 2010; Yamaji et al., 2010). Our analyses showed that TaeEF1A is also an important host factor for CWMV, BSMV, or WYMV infection in wheat plants (Figure 1a). In addition, the expression of NbeEF1A was upregulated under CWMV, TMV, or TuMV infection in *N. benthamiana* (Figure 1b). These results suggest that eEF1A may be a general host factor required for infection by different viruses. eEF1A is known to be highly conserved throughout the eukaryotes. Our amino acid sequence analysis also showed that eEF1As in *N. benthamiana* and *T. aestivum* are highly conserved (Figure S1), suggesting that these two eEF1As share similar properties. *N. benthamiana* is an excellent experimental host plant for studies of virus infections, including rice stripe virus (RSV), bamboo mosaic virus (BaMV), and BSMV (Alazem et al., 2017; Shi et al., 2016; Zhang et al., 2018). Thus, we reasoned that *N. benthamiana* would be a suitable host plant for studying the effect of NbeEF1A during CWMV infection. Indeed, we also demonstrated that the expression of NbeEF1A was positively correlated with the accumulation of CWMV CP and genomic RNAs during CWMV infection from 7 to 28 dpi (Figure 1c–e). When *NbeEF1A* expression in *N. benthamiana* plants was silenced through VIGS, we found that accumulation of CWMV CP and genomic RNAs was significantly reduced (Figure 2). By contrast, transient overexpression of *NbeEF1A* in *N. benthamiana* plants significantly increased the accumulation of CWMV CP and genomic RNAs (Figure 3a,b). We also found that the replication and translation efficiency of CWMV exhibited significant changes when *NbeEF1A* was silenced or overexpressed in *N. benthamiana* plants (Figures 2d,e and 3c,d). Based on these findings, we conclude that eEF1A is required for CWMV infection in plants. To our knowledge, this is the first report to show that eEF1A regulates CWMV infection in plants.

Virus replication complexes (VRC) are sites where viral RNAs are synthesized in cells (Cotton et al., 2009). Previous studies have reported that eEF1A is a component of VRCs and can control the replication of different viruses via interactions with viral RdRps (Thivierge et al., 2008; Yamaji et al., 2006). In this study, we found that NbeEF1A cannot interact with CWMV RdRp in vivo or in vitro (Figure 4b–d). VRCs are known to contain ribosomes, tubulin‐like structures, endoplasmic reticulum, RdRp and other viral proteins, and viral RNAs (Asurmendi et al., 2004; Liu et al., 2005). Moreover, eEF1A has been shown to form complexes with RdRp and other proteins encoded by TuMV (Thivierge et al., 2008) or to help VRC formation through interactions with viral VPg‐Pro (Thivierge et al., 2008). Although eEF1A does not interact directly with CWMV RdRp, it may nonetheless promote CWMV multiplication through its participation in VRC formation. Shamovsky et al. have shown that eEF1A can bind to aminoacylated tRNAs and other mammalian RNA species (Shamovsky et al., 2006). In a separate report, Zeenko et al. have shown that eEF1A can interact with the 3ʹ‐UTR of TMV genomic RNA to regulate virus replication in *N. benthamiana* (Zeenko et al., 2002). TYMV and BMV also have TLSs in their RNAs and these TLSs can all bind to eEF1A to promote viral replication (Annamalai & Rao, 2007; Matsuda & Dreher, 2004; Matsuda et al., 2004). These previous reports encouraged us to investigate whether NbeEF1A can bind to the TLSs in UTRs of CWMV RNAs. Our results show that NbeEF1A is indeed capable of interacting with the 3ʹ‐UTR of CWMV genomic RNAs (Figure 4e). Several studies have also shown that eEF1A can bind to RNA structures outside 3ʹ‐UTRs. For example, eEF1A has been shown to bind to a region in the Hepatitis delta virus genomic RNA to promote HDAg mRNA transcription and the synthesis of its negative‐strand RNA (Sikora et al., 2009). The 5ʹ‐UTR of HIV‐1 genomic RNA has been shown to interact with eEF1A and this interaction is important for late DNA synthesis during reverse transcription (Li et al., 2015). It has been demonstrated that for some plant RNA viruses the VRC could specifically recognize the 3ʹ‐terminal portion of the viral genomic RNA, which contains a unique promoter to enhance viral replication (Osman et al., 2000; Vlot et al., 2001). The specific binding of NbeEF1A to the 3ʹ‐UTR of CWMV genomic RNAs (Figure 5) suggests a role for eEF1A‐binding in both assembly of the CWMV VRC and template recognition for viral replication. However, other potential roles of the eEF1A interaction to CWMV RNA 3ʹ‐UTR, such as anchoring the CWMV VRC to a specific membrane or cytoskeleton in a host cell or participation in cell‐to‐cell spread of viral RNA, should also be examined in future studies. Taken together, our results show that the 3ʹ‐UTR of CWMV genomic RNAs is required for CWMV RNA replication.

The 3ʹ‐UTRs often are composed of SLs, pseudoknots (PKs), or TLSs, and tend to be conserved among different RNA viruses and within virus groups. Here, we also used the RNA structure Web to predict the PKs in the 3ʹ‐UTR of RNA1 and RNA2 of CWMV. The results showed that the 3ʹ‐UTR of RNA1, but not of RNA2, contains a potential PK (Figure S6c). Several studies have also shown that PKs in the TLS of turnip yellow mosaic virus, BMV, and TMV are involved in infectivity of these viruses (Chandrika et al., 2000; Deiman et al., 1998; Dreher et al., 1996). The PK region and TL regions interact with eEF1A independently or simultaneously during TMV infection (Zeenko et al., 2002). Thus, we speculate that the potential PKs may be responsible for the weak infectivity of CWMV ΔR1 (Figure 6c, d), which also requires further experimental analysis in the future. MST analysis also showed that the TLS in the CWMV 3ʹ‐UTR is essential for the binding between NbeEF1A and CWMV genomic RNAs (Figure 6a). The binding ability of CWMV RNA with a mutagenized 3ʹ‐UTR showed a 65% reduction compared with the WT CWMV RNA, possibly due to the weak affinity of eEF1A for RNA. Thus, eEF1A binding to the CWMV 3ʹ‐UTR might be required for both the initiation of viral negative‐strand RNA synthesis and enhancing viral replication. Secondary structures in viral RNAs play pivotal roles in viral life cycles. The secondary or more advanced structures in virus genomic RNAs can stabilize RNAs and allow specific interactions between different RNA molecules or between RNAs and proteins. An early investigation of turnip yellow mosaic virus showed that the TLS at the 3ʹ end of viral RNA participates in virus replication (Matsuda & Dreher, 2004). Here, six TSL structures were predicted in the CWMV RNA2 3ʹ‐UTR (Figure S6). Our mutational analyses showed that SL‐6 was important for binding eEF1A and the CWMV 3ʹ‐UTR (Figure 7). Because the affinity of eEF1A for viral RNA is independent of GTP and other RNA binding sites that are specific for binding to aa‐tRNA (Slobin, 1983), we considered that the binding of eEF1A to the SL‐6 of the CWMV 3ʹ‐UTR plays an important role in CWMV infection. To confirm this idea, we generated a series of CWMV mutants with an altered SL‐6 (CWMV^ugaacau^), a deleted SL‐6 (CWMV^Δ3561–3567^), and a compensatory SL‐6 (CWMV^InsACCGGCC^). *N. benthamiana* plants inoculated with either the CWMV^ugaacau^ or the CWMV^Δ3561–3567^ mutant virus accumulated significantly lower levels of viral RNA and protein than plants inoculated with WT CWMV, whereas plants inoculated with the CWMV^InsACCGGCC^ mutant virus, which had a restored stem structure, accumulated similar levels of viral RNA and protein to that of the WT CWMV (Figure 8). Mutation in the TLS can affect RNA affinity for eEF1A (Matsuda & Dreher, 2004). This finding suggests that the mutation in SL‐6 of CWMV 3ʹ‐UTR interferes with its ability to bind to eEF1A and disrupts the synthesis of the CWMV negative‐strand, thereby impeding the initiation of viral RNA replication.

eEF1A is one of the most abundant proteins in eukaryotic cells and is one of the most characterized proteins of the translational machinery (Andersen et al., 2003). Our results have demonstrated that eEF1A is involved in the replication and translation of CWMV through binding to the CWMV 3ʹ‐UTR. Thus, it seems likely that CWMV might have evolved to use it in various ways. Future studies should reveal the relevance of eEF1A not only for CWMV replication but also for translation or other CWMV infection steps, which should contribute to our understanding of host–virus interactions, propagation strategies, and the adaptive evolution of RNA viruses.

## 4 EXPERIMENTAL PROCEDURES

### 4.1 Plant growth and virus inoculation

*N. benthamiana* plants were grown inside a growth chamber maintained at 25 °C and a 14 hr light/10 hr dark cycle. *Agrobacterium tumefaciens* GV3101 harbouring CWMV RNA1 (pCB‐35S‐R1) or RNA2 (pCB‐35S‐R2) infectious clones were obtained from a previously reported source (Yang et al., 2016). The mutant CWMV RNA2 constructs produced in this study were also transformed individually into *A. tumefaciens* GV3101, and the *Agrobacterium* cultures were grown individually overnight at 28 °C in a yeast extract peptone medium containing kanamycin (50 µg/ml) and rifampicin (50 µg/ml). The resulting *Agrobacterium* cultures were pelleted and then resuspended in an infiltration buffer (100 mM MES, pH 5.2, 10 mM MgCl_2_, 200 mM acetosyringone) to obtain an OD_600_ of 0.6 followed by >2 hr incubation at 25 °C. *Agrobacterium* harbouring pCB‐35S‐R1 was mixed with *Agrobacterium* harbouring pCB‐35S‐R2 in a 1:1 ratio or with one of its derivatives prior to infiltration into *N*. *benthamiana* leaves. The infiltrated plants were grown inside a growth chamber maintained at 15 ± 2 °C, with a 14 hr/10 hr (light/dark) cycle and 70% relative humidity.

Wheat cv. Yangmai 158 plants were grown to the two‐leaf stage in a greenhouse and then inoculated with in vitro transcripts of CWMV, BSMV, or WYMV after adding 1 vol of FES buffer (1% wt/vol sodium pyrophosphate, 1% wt/vol macaloid, 1% wt/vol celite, 0.5 M glycine, 0.3 M K_2_HPO_4_, pH 8.5, with phosphoric acid). Inoculum was applied by gently rubbing it on the surface of leaves that had been abraded with carborundum.

### 4.2 Plasmid construction

Full‐length *TaeEF1A* or *NbeEF1A* sequences were RT‐PCR amplified from a *T. aestivum* or a *N. benthamiana* leaf sample, respectively. The resulting PCR products were purified using a Gene JET gel extraction kit (Thermo Fisher Scientific) and cloned individually into a pGW‐B5C vector (Invitrogen) to produce p35S:TaeEF1A and p35S:NbeEF1A using Gateway cloning technology and following the manufacturer's instructions (Invitrogen).

For VIGS, a partial sequence of *NbeEF1A* (nucleotide positions 1451–1750) was RT‐PCR amplified, double digested with *Bam*HI *and Sma*I restriction enzymes (New England Biolabs) and cloned into the vector pTRV (Liu et al., 2002), a TRV RNA2‐based vector, to produce pTRV:NbeEF1A. *Agrobacterium* harbouring the pYL196 vector (i.e., the TRV RNA1‐based vector) was mixed with an equal amount of *Agrobacterium* harbouring the pTRV:NbeEF1A vector (the mixed *Agrobacterium* is referred to as TRV:NbeEF1A hereafter) and infiltrated into *N. benthamiana* leaves.

To determine the effect of SL structures in the CWMV 3ʹ‐UTR on viral replication, we deleted the SL‐6 from the 3ʹ‐UTR of CWMV RNA2 or altered its SL structure by replacing ACCGGCC with ugaacau to produce mutants 3ʹ‐UTR^Δ3561–3565^ or m3ʹ‐UTR^ugaacau^, respectively. The mutant 3ʹ‐UTR^Δ3561–3565^ was made through RT‐PCR amplification using specific primers from a CWMV‐infected *N. benthamiana* leaf sample, and mutants m3ʹ‐UTR^ugaacau^ and m3ʹ‐UTR^InsACCGGCC^ were synthesized by Sangon Biotech, Hangzhou, Zhejiang, China. The resulting sequences were digested with *Bam*HI and *Sma*I restriction enzymes and cloned individually into the pUC1301 vector.

For prokaryotic expression of the NbeEF1A protein, the full‐length *NbeEF1A* gene was RT‐PCR amplified, digested with *Bam*HI and *Sma*I restriction enzymes, and cloned into pET32a (Novagen) to produce pET:NbeEF1A.

For the yeast two‐hybrid assay, full‐length *TaeEF1A* and *NbeEF1A* sequences were RT‐PCR amplified, digested with *Nde*I and *Bam*HI restriction enzymes, and cloned individually into pGBKT7 to produce pBD:TaeEF1A and pBD:NbeEF1A, respectively. Three segments encoding three different CWMV replicase regions, amino acid positions 1–670, 670–1430, and 1430–1840, were PCR amplified and cloned into the pGADT7 vector to produce pAD‐Rep^1–670^ (Met), pAD‐Rep^670–1430^ (Hel), and pAD‐Rep^1430–1840^ (RdRp), respectively. The construction of these three activation domain vectors has been described previously (Yang et al., 2017). The primers used in this study are listed in Table S1.

### 4.3 Silencing *NbeEF1A* expression through virus‐induced gene silencing

To silence *NbeEF1A* expression in *N. benthamiana* plants, leaves were infiltrated with *Agrobacterium* harbouring TRV:NbeEF1A. The VIGS procedure used in this study was similar to that reported previously (Ratcliff et al., 2001). The TRV:NbeEF1A‐ or TRV:00‐infected (control) plants were grown at 25 °C for 7 days and then inoculated again with CWMV through agroinfiltration and maintained at 15 °C.

### 4.4 RNA extraction and RT‐qPCR analysis

Total RNA was extracted from *T. aestivum* and *N. benthamiana* leaf samples with a HiPure plant RNA mini kit (Magen). First‐strand cDNA was synthesized using a first‐strand cDNA synthesis kit (TOYOBO) followed by quantitative PCR using an Applied Biosystems QuantStudio 6 Flex system (Applied Biosystems) and a SYBR Green Master Mix kit (Vazyme). Each treatment had three biological replicates and each biological replicate had four technical replicates. The relative expression levels of assayed genes or the CWMV *CP* gene were calculated using the 2^−ΔΔ^ *^C^* ^t^ method (Livak & Schmittgen, 2001). The primers used in this study are listed in Table S1.

### 4.5 Northern blot assays

Northern blot assays were performed as previously described (Yang et al., 2016). Briefly, total RNA (c.5 µg per sample) was separated in a 2% formaldehyde agarose gel through electrophoresis (60 V for 1.5 hr). The separated RNA bands were transferred onto a Hybond‐N^+^ membrane (Amersham Biosciences) followed by a 10 min crosslink under a UV light. The blot was hybridized with a digoxigenin‐labelled probe specific for the 3ʹ end of CWMV genomic RNAs. The probe was produced using a Detection Starter Kit II following the manufacturer's instructions (Roche).

### 4.6 Western blot assays

Western blot assays were conducted as previously described (Yang et al., 2016). Protein samples were separated using SDS‐PAGE and transferred onto nitrocellulose membranes. Blots were blocked using phosphate‐buffered saline (PBS) containing 5% skimmed milk, rinsed several times with PBS, and then probed with a specific primary mouse antibody (at a 1:5,000 dilution) followed by an anti‐mouse (at a 1:10,000 dilution) alkaline phosphatase conjugate. The detection signal was visualized by incubating blots in an enhanced chemiluminescence solution according to the manufacturer's instructions (Invitrogen) and scanned using an Amersham Imager 680 machine (GE Healthcare BioSciences).

### 4.7 Translation efficiency assay

Translation efficiency was assayed as previously described with minor modifications (Merchante et al., 2015). First, 10 g of *N. benthamiana* leaves was ground into fine powder in liquid nitrogen. Then, 2 g of sample was used to extract the total RNA with a HiPure plant RNA mini kit (Magen). Next, the other 8 g of sample was suspended in 20 ml of polysome extraction buffer (200 mM Tris‐HCl, pH 9.0, 35 mM MgCl_2_, 200 mM KCl, 25 mM EGTA, 1% vol/vol Triton X‐100, 1% vol/vol IGEPAL CA‐630, 5 mM dithiothreitol [DTT], 1 mM phenylmethylsulfonyl fluoride [PMSF], 50 μg/ml chloramphenicol, 100 μg/ml cycloheximide) at 4 °C for 20 min with slight shaking. The mixture was centrifuged twice at 16,000 × g for 20 min at 4 °C. Then, 16 ml of supernatant was slowly transferred onto 15 ml of sucrose buffer (1.75 M sucrose, 400 mM Tris‐HCl, pH 9.0, 35 mM MgCl_2_, 5 mM EGTA, 200 mM KCl, 5 mM DTT, 50 μg/ml chloramphenicol, 50 μg/ml cycloheximide). After centrifugation at 200,000 × g for 4 hr at 4 °C, the supernatant was carefully removed and the polysomes in the bottom were resuspended in 300 μl of diethylpyrocarbonate [DEPC]‐treated water. The polysomes RNA was isolated using TRIzol reagent (Life Technologies) and then subjected to reverse transcription and real‐time PCR analysis.

### 4.8 Yeast two‐hybrid assay

The yeast two‐hybrid assay was performed using a Matchmaker Gold yeast two‐hybrid system and a Yeastmaker yeast transformation system 2 according to the manufacturer's instructions (Clontech Laboratories).

### 4.9 In vitro transcription

PCR products containing a T7 promoter followed by a specific probe sequence (i.e., sense or antisense [+/−] CWMV 3ʹ‐UTR, [+/−] CWMV 5ʹ‐UTR, and [+/−] CWMV^301–781^) were prepared using a T7 in vitro transcription kit following the manufacturer's instructions (Thermo Fisher Scientific) and used as unlabelled (UL) RNA probes.

### 4.10 Electrophoretic mobility shift assay

Biotin was integrated at the 3ʹ end of the UL RNA probes using a Pierce RNA 3ʹ end biotinylation kit (Thermo Fisher Scientific) to produce BL RNA probes. The EMSA binding reaction and chemiluminescence detection were conducted using a LightShift chemiluminescent RNA EMSA (REMSA) kit (Thermo Fisher Scientific). Briefly, a BL RNA probe was incubated with a purified protein sample for 30 min at 25 °C. The mixture was analysed in a 5% native polyacrlamide gel by performing electrophoresis, transferred onto a Hybond‐N^+^ membrane, and crosslinked for 45 s under an UV light. The detection signal was scanned using an Amersham Imager 680 machine.

### 4.11 Microscale thermophoresis assay

The affinity of the purified NbeEF1A for RNA was determined using Monolith NT.115 (NanoTemper Technologies). Microscale thermophoresis (MST) labelling of NbeEF1A was conducted in PBS solution containing a Monolith NT protein labelling kit RED according to the manufacturer's instructions (NanoTemper Technologies). Samples were then loaded into NanoTemper hydrophilic‐treated capillaries. The resulting samples were analysed by the manufacturer using NanoTemper analytical software to estimate their equilibrium dissociation constant (*K*_D_) values.

### 4.12 Pull‐down

Purified GST–NbeEF1A protein was incubated with purified His, TuMV VPg‐Pro‐His, CWMV Met‐His, CWMV Hel‐His, or CWMV RdRp‐His at room temperature for 15 min. Next, 25 μl of GST‐Trap agarose (ChromoTek) was added to each reaction system, which was then incubated at 4 °C for 2 hr. After beads were collected and washed three times with Tris‐buffered saline solution (TBS; 10 mM Tris‐HCl pH 8.0, 150 mM NaCl), the reaction mixtures were run using SDS‐PAGE and immunoblotted with an anti‐GFP antibody (TransGene) and an anti‐His antibody (TransGene).

### 4.13 Co‐immunoprecipitation assay

Proteins were transiently coexpressed in leaves of *N. benthamiana* by *Agrobacterium* infiltration. Coimmunoprecipitation assays were performed on *N. benthamiana* leaves that were harvested 2 days after infiltration, pooled, and ground in liquid nitrogen. Total protein was extracted with an extraction buffer (50 mM Tris‐HCl pH 7.5, 150 mM NaCl, 10 mM MgCl_2_, 5 mM DTT, 0.1% Triton X‐100). Protein extracts were incubated with 25 μl GFP‐Trap agarose (ChromoTek) for immunoprecipitation ranging from 2 hr to overnight at 4 °C. Finally, beads were collected and washed three times with TBS solution, then the reaction mixtures were run using SDS‐PAGE and immunoblotted with an anti‐GFP antibody and an anti‐His antibody.

## CONFLICT OF INTEREST

The authors declare that there is no conflict of interest.

## AUTHOR CONTRIBUTIONS

J.Y., H.Z., and J.C. conceived the project and designed the experiments. X.C. carried out the experiments with assistance from M.X., L.H., J.L., T.Z., J.Y., and Q.L. All authors analysed and discussed the results, and J.Y. wrote the manuscript. All authors confirm that they have no conflict of interest to declare.

## Supporting information


**FIGURE S1** Amino acid homology‐based analysis using nine different eEF1As. Sequences of *Capsicum annuum* (*Ca*), *Solanum lycopersicum* (*Sl*), *Solanum pennellii* (*Sp*), *Solanum tuberosum* (*St*), *Ipomoea nil* (*In*), *Oryza sativa* (*Os*), *Tarenaya hassleriana* (*Th*), *Triticum aestivum* (*Ta*), and *Nicotiana benthamiana* (*Nb*) eEF1As were retrieved from the National Center for Biotechnology Information (NCBI) (https://www.ncbi.nlm.nih.gov/) and aligned using DNAMAN v. 6.0 (Lynnon Biosoft Corp.)

**FIGURE S2** Analyses of *NbeEF1A* expression in silenced plants. Quantitative reverse transcription PCR was used to determine the relative expression of *NbeEF1A* in TRV:00 or TRV:NbeEF1A‐inoculated *Nicotiana benthamiana* plants. The expression level of the *Actin* gene was used as an internal control. Values shown are the mean ± *SD* of three biological replicates; each biological replicate had three technical replicates. *p < .05; n.s., no significant difference based on Student’s *t* test

**FIGURE S3** Detection of NbeEF1A expression. Western blot assay using an anti‐GFP antibody. Tissues were harvested from GFP‐ or NbeEF1A‐GFP‐inoculated leaves of *Nicotiana benthamiana* plants. Coomassie Brilliant blue (CBB) staining was used to visualize sample loadings

**FIGURE S4** Analysis of interactions between TaeEF1A and different regions of CWMV RdRp. The right‐hand column shows that yeast cells co‐expressing BD:TaeEF1A and AD:Replicase, BD:TaeEF1A and AD:Met, BD:TaeEF1A and AD:Hel, or BD:TaeEF1A and AD:RdRp grow well on SD/−Trp−Leu selective medium. The left‐hand column shows that these yeast cells could not grow on SD/−Trp−Leu−His−Ade selective medium, indicating no positive reactions. Yeast cells co‐expressing BD‐53 and AD‐T or BD‐Lam and AD‐T acted as positive and negative controls, respectively

**FIGURE S5** Electrophoretic mobility shift assays for NbeEF1A binding with different segments of CWMV RNA1 or RNA2. (a) NbeEF1A binding activity for (−) 3ʹ‐UTR, (+) 5ʹ‐UTR, (−) 5ʹ‐UTR, (+) CWMV^301–781^ or (−) CWMV^301–781^ RNA of RNA1. (b) NbeEF1A binding activity for (−) 3ʹ‐UTR, (+) 5ʹ‐UTR, (−) 5ʹ‐UTR, (+) CWMV^301–781^ or (−) CWMV^301–781^ RNA of RNA2. Each treatment has three components: a biotin‐labelled (BL) RNA probe, an unlabelled (UL) RNA probe (UL‐RNA corresponds to labelled RNA), and a purified recombinant NbeEF1A or other protein. BL‐RNA + NbeEF1A is used to show binding and UL‐RNA + BL‐RNA + NbeEF1A is used to show competitive binding. Bovine serum albumen (BSA) and green fluorescent protein (GFP) acted as negative controls

**FIGURE S6** Nucleotide sequence analysis and secondary structure predictions. (a) Analysis of nucleotide sequences representing the 3ʹ‐UTR of CWMV genomic RNA1 and RNA2. (b) Prediction of secondary structures in of the CWMV genomic RNA1 and RNA2. (c) Pseudoknot structure of the CWMV RNA1 3ʹ‐UTR. This structure is derived from RNA structure Web

**TABLE S1** Primers used for vector constructions, quantitative PCR, and reverse transcription PCR

## Data Availability

The data that support the findings of this study are available from the corresponding author upon reasonable request.
