# Supplementary figures and images for: Binding between elongation factor 1A and the 3ʹ‐UTR of Chinese wheat mosaic virus is crucial for virus infection
Source: Mol Plant Pathol. 2021 Aug 17;22(11):1383–98. doi: 10.1111/mpp.13120 (PMC8518580; doi:10.1111/mpp.13120)

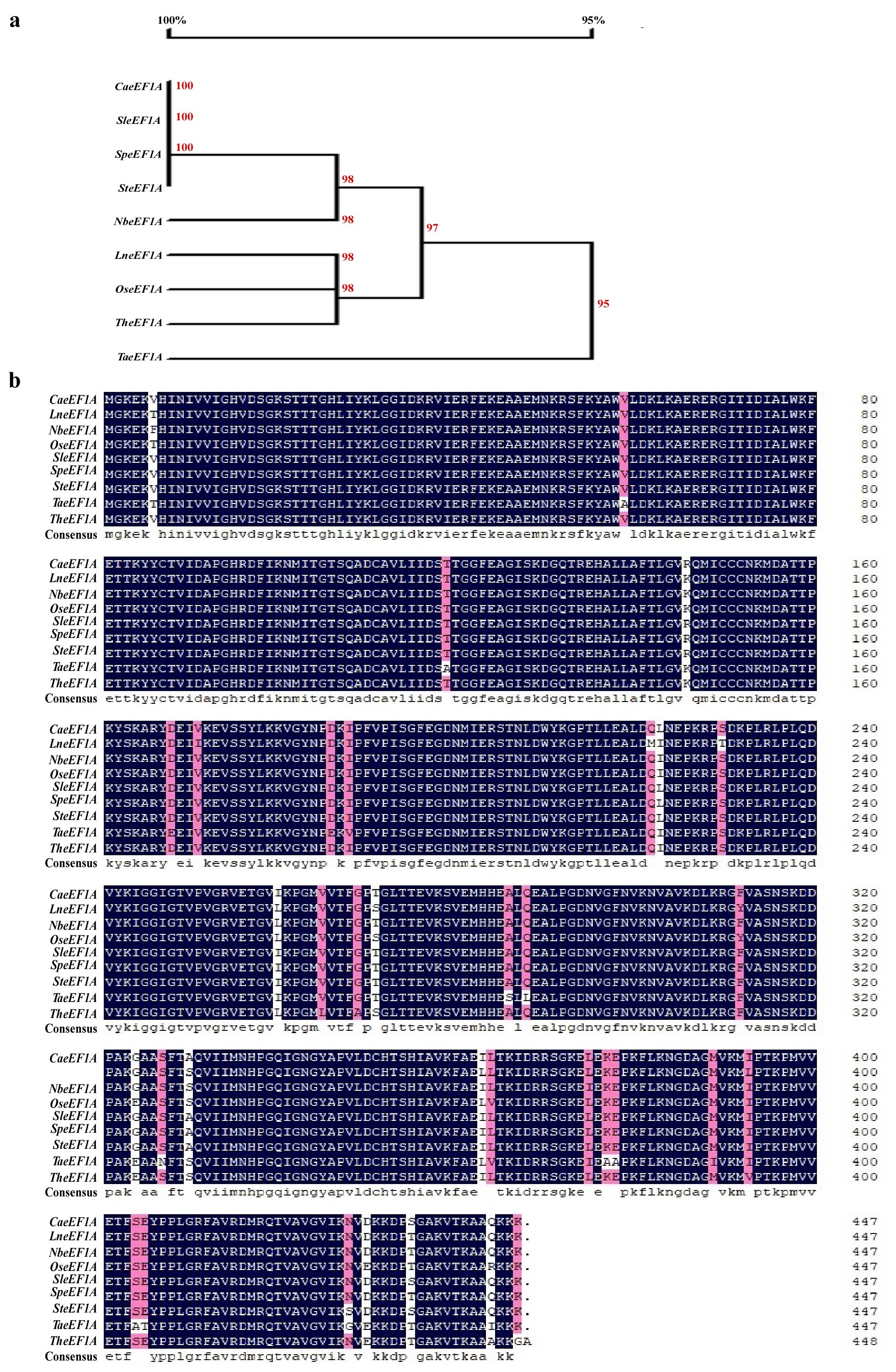

Supplement: Supplementary file 1 — FIGURE S1 Amino acid homology‐based analysis using nine different eEF1As. Sequences of Capsicum annuum (Ca), Solanum lycopersicum (Sl), Solanum pennellii (Sp), Solanum tuberosum (St), Ipomoea nil (In), Oryza sativa (Os), Tarenaya hassleriana (Th), Triticum aestivum (Ta), and Nicotiana benthamiana (Nb) eEF1As were retrieved from the National Center for Biotechnology Information (NCBI) (https://www.ncbi.nlm.nih.gov/) and aligned using DNAMAN v. 6.0 (Lynnon Biosoft Corp.) [file MPP-22-1383-s003.jpg]

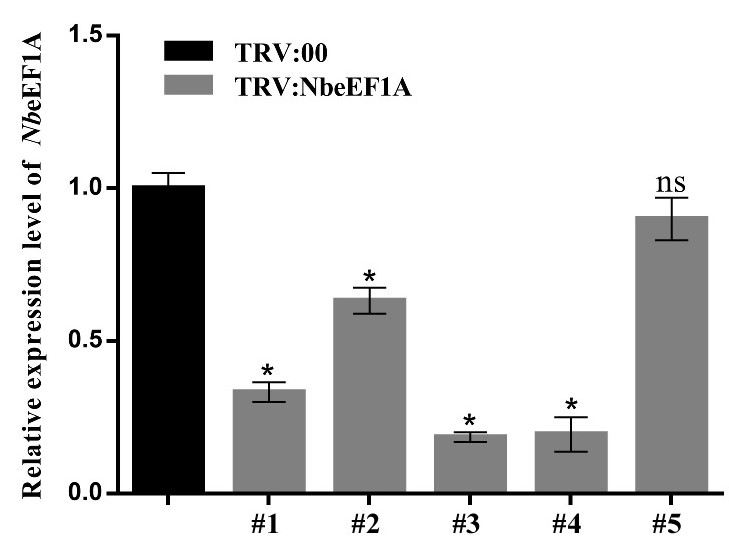

Supplement: Supplementary file 2 — FIGURE S2 Analyses of NbeEF1A expression in silenced plants. Quantitative reverse transcription PCR was used to determine the relative expression of NbeEF1A in TRV:00 or TRV:NbeEF1A‐inoculated Nicotiana benthamiana plants. The expression level of the Actin gene was used as an internal control. Values shown are the mean ± SD of three biological replicates; each biological replicate had three technical replicates. *p < .05; n.s., no significant difference based on Student’s t test [file MPP-22-1383-s006.jpg]

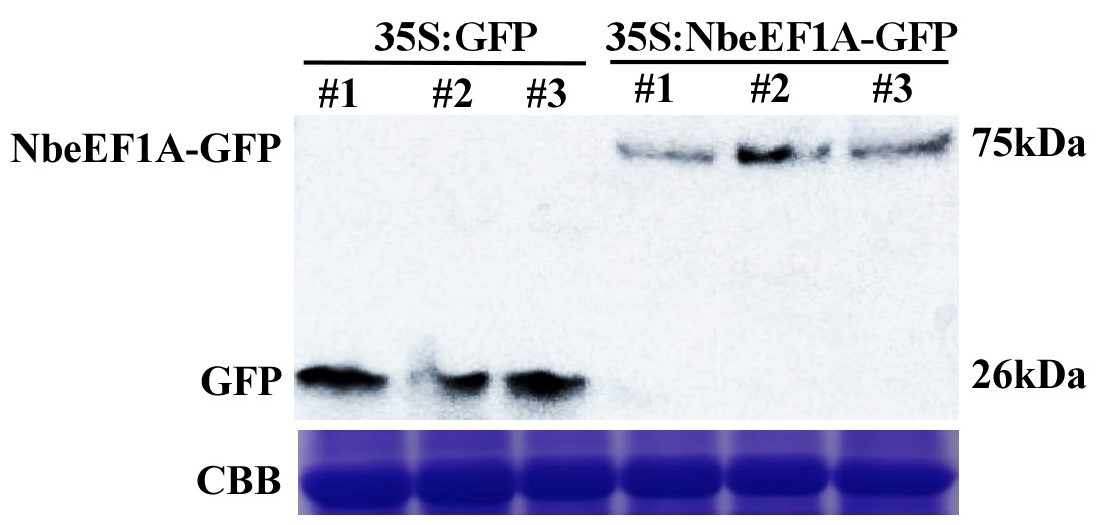

Supplement: Supplementary file 3 — FIGURE S3 Detection of NbeEF1A expression. Western blot assay using an anti‐GFP antibody. Tissues were harvested from GFP‐ or NbeEF1A‐GFP‐inoculated leaves of Nicotiana benthamiana plants. Coomassie Brilliant blue (CBB) staining was used to visualize sample loadings [file MPP-22-1383-s004.jpg]

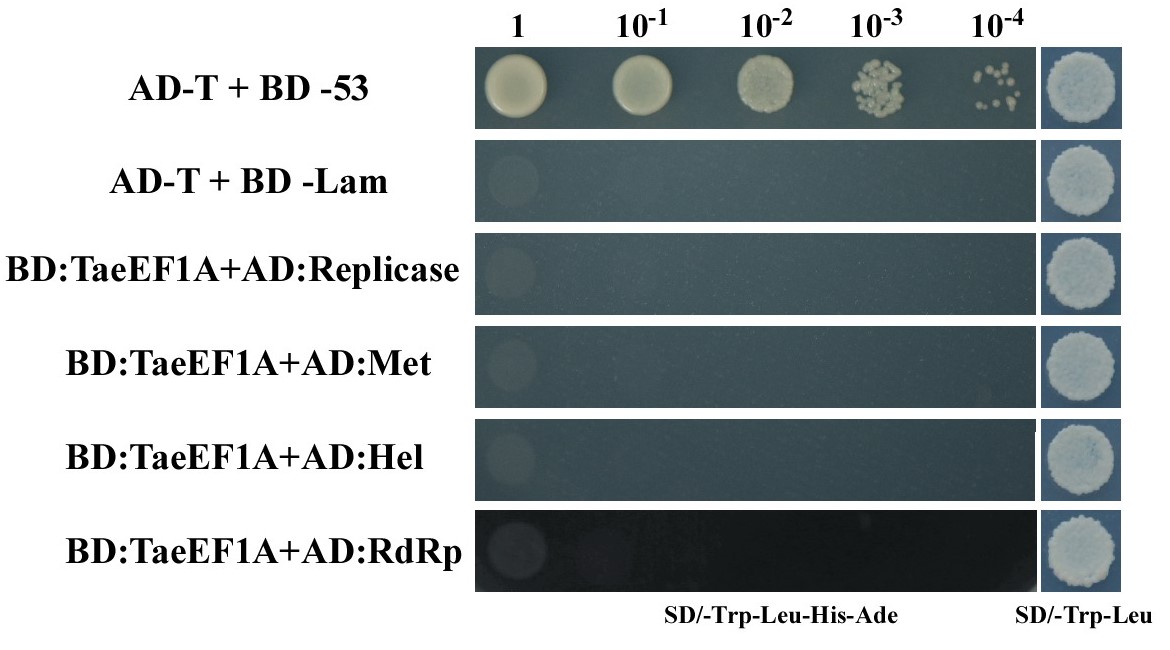

Supplement: Supplementary file 4 — FIGURE S4 Analysis of interactions between TaeEF1A and different regions of CWMV RdRp. The right‐hand column shows that yeast cells co‐expressing BD:TaeEF1A and AD:Replicase, BD:TaeEF1A and AD:Met, BD:TaeEF1A and AD:Hel, or BD:TaeEF1A and AD:RdRp grow well on SD/−Trp−Leu selective medium. The left‐hand column shows that these yeast cells could not grow on SD/−Trp−Leu−His−Ade selective medium, indicating no positive reactions. Yeast cells co‐expressing BD‐53 and AD‐T or BD‐Lam and AD‐T acted as positive and negative controls, respectively [file MPP-22-1383-s001.jpg]

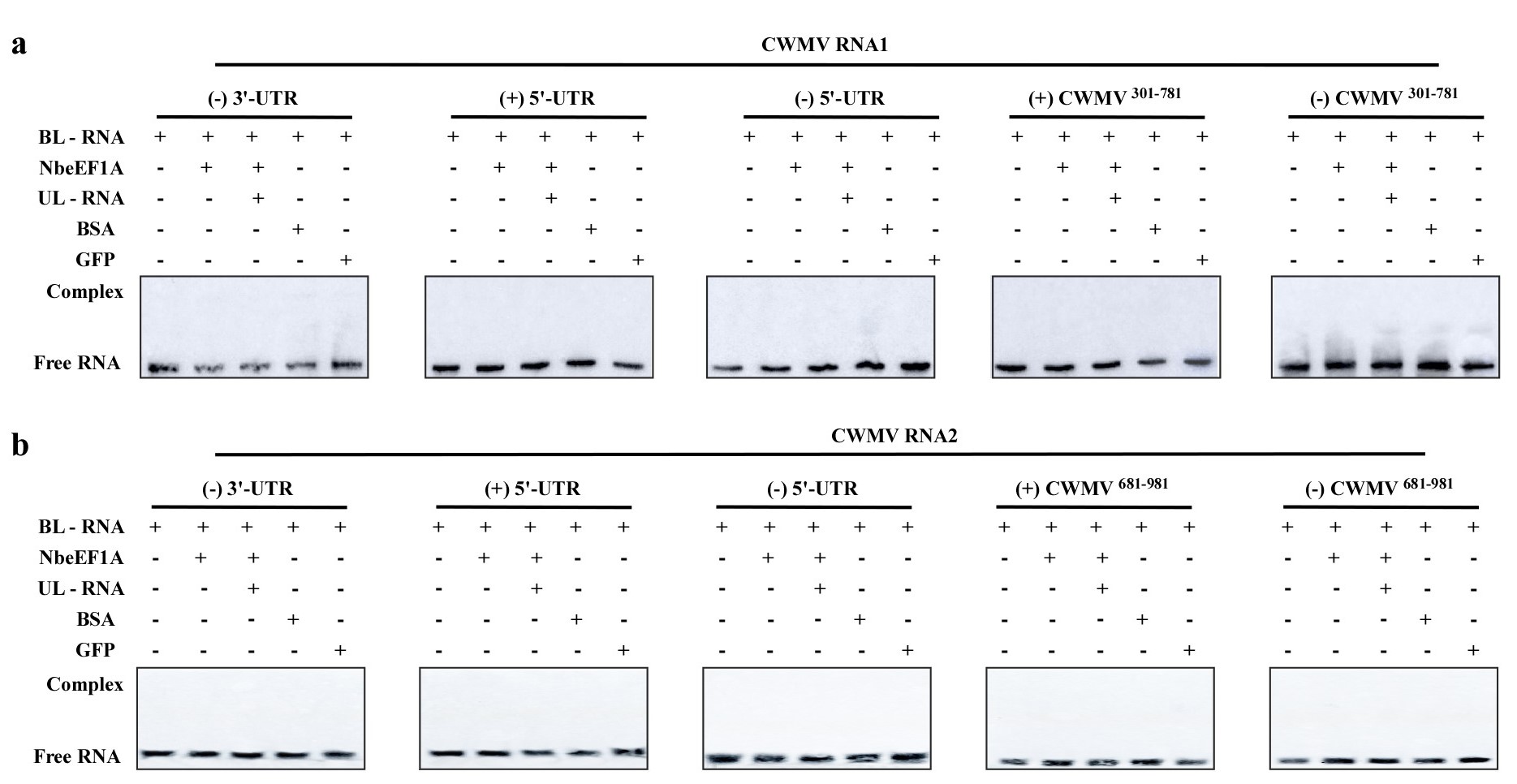

Supplement: Supplementary file 5 — FIGURE S5 Electrophoretic mobility shift assays for NbeEF1A binding with different segments of CWMV RNA1 or RNA2. (a) NbeEF1A binding activity for (−) 3ʹ‐UTR, (+) 5ʹ‐UTR, (−) 5ʹ‐UTR, (+) CWMV301–781 or (−) CWMV301–781 RNA of RNA1. (b) NbeEF1A binding activity for (−) 3ʹ‐UTR, (+) 5ʹ‐UTR, (−) 5ʹ‐UTR, (+) CWMV301–781 or (−) CWMV301–781 RNA of RNA2. Each treatment has three components: a biotin‐labelled (BL) RNA probe, an unlabelled (UL) RNA probe (UL‐RNA corresponds to labelled RNA), and a purified recombinant NbeEF1A or other protein. BL‐RNA + NbeEF1A is used to show binding and UL‐RNA + BL‐RNA + NbeEF1A is used to show competitive binding. Bovine serum albumen (BSA) and green fluorescent protein (GFP) acted as negative controls [file MPP-22-1383-s002.jpg]

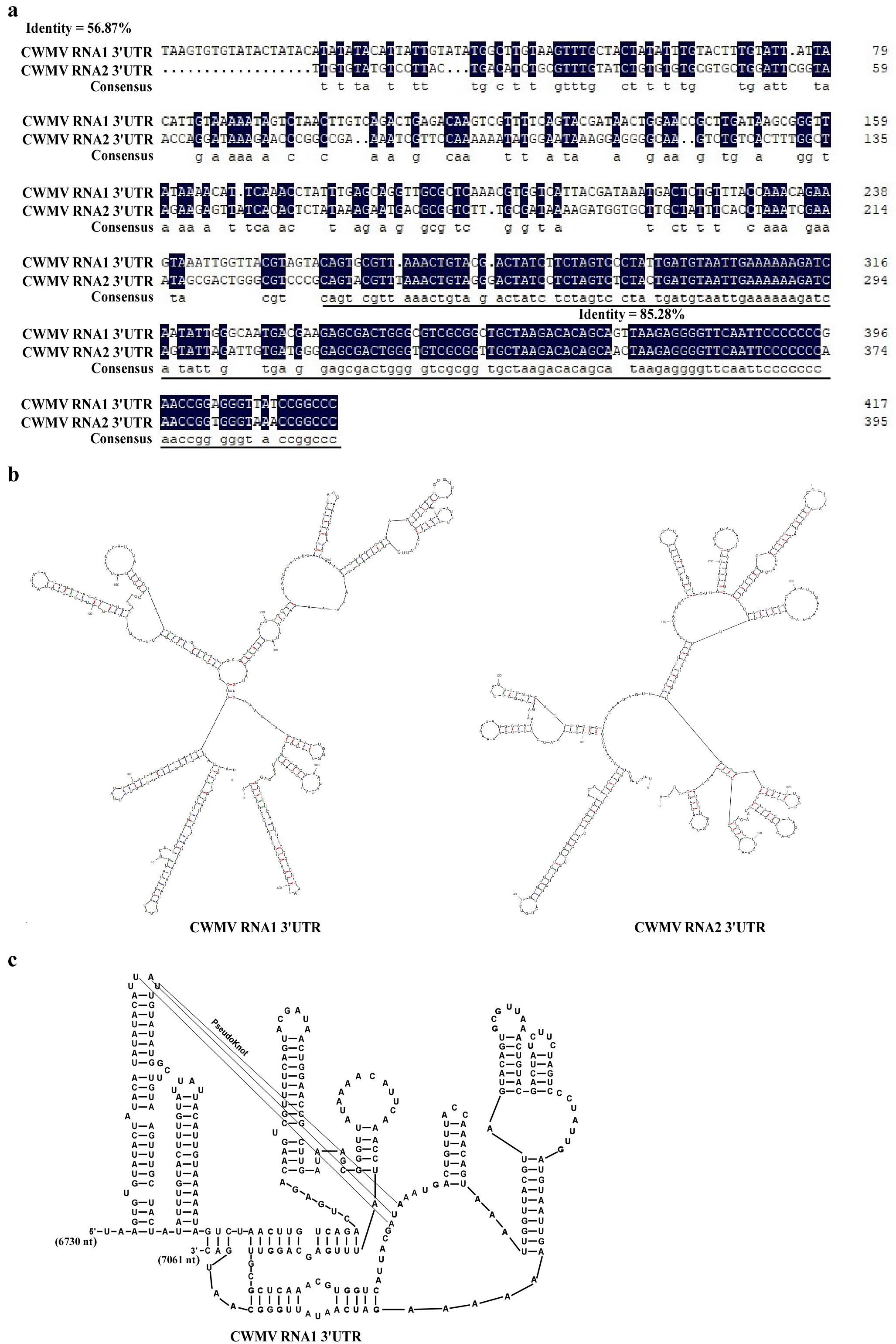

Supplement: Supplementary file 6 — FIGURE S6 Nucleotide sequence analysis and secondary structure predictions. (a) Analysis of nucleotide sequences representing the 3ʹ‐UTR of CWMV genomic RNA1 and RNA2. (b) Prediction of secondary structures in of the CWMV genomic RNA1 and RNA2. (c) Pseudoknot structure of the CWMV RNA1 3ʹ‐UTR. This structure is derived from RNA structure Web [file MPP-22-1383-s005.jpg]
