# Supplementary material for: Binding between elongation factor 1A and the 3ʹ‐UTR of Chinese wheat mosaic virus is crucial for virus infection
Source: Mol Plant Pathol. 2021 Aug 17;22(11):1383–98. doi: 10.1111/mpp.13120 (PMC8518580; doi:10.1111/mpp.13120)
Supplement: Supplementary file 7 — TABLE S1 Primers used for vector constructions, quantitative PCR, and reverse transcription PCR [file MPP-22-1383-s007.docx]

**Table S1. Primers used in vector construction and molecular analyses**

| **Primers** | **Primer sequences (5'-3')^a^** | **Position and description ^b^** | **Usage** |
| --- | --- | --- | --- |
| *Ta*actinF | CTAACTGACTGTTTGATGAAG | Partial sequence of *Ta*actin nt 301-322 | qRT-PCR of *Ta*actin |
| *Ta*actinR | CTTCATGATAGAGTTGTAGGT | Partial sequence of *Ta*actin nt 579-600 |  |
| *Nb*Actin-F | AGGCTGTTCTTTCCCTCTATGC | Partial sequence of *Nb*actin nt 121-142 | qRT-PCR of *Nb*actin |
| *Nb*Actin-R | CAACTTCTCCTTCACATCCCTAAC | Partial sequence of *Nb*actin nt 399-420 |  |
| Oliga DT | TTTTTTTTTTTTTTTTTTTTTTTT |  |  |
| Attb1 | GGGG ACAAGTTTGTACAAAAAAGCAGGCTGC |  |  |
| Attb2 | GGGGACCACTTTGTACAAGAAAGCTGGGTC |  |  |
| P1-N | ATCTCAGAGGAGGACCTGCATATGATGGGTAAAGAGAAATTTCAC | *NdeⅠ*, partial sequence of *Nb*eEF1A nt 123-143 | BD:*Nb*eEF1A construction |
| P1-C | TGCGGCCGCTGCAGGTCGACGGATCCCTCATTTCTTCTTCTGAGCAGC | *BamHⅠ*, partial sequence of *Nb*eEF1A nt 1494-1473 |  |
| P2-N | ATCTCAGAGGAGGACCTGCATATGATGGGTAAGGAGAAGACTCAC | *NdeⅠ*, partial sequence of *Nb*eEF1A nt 131-151 | BD:*Ta*eEF1A construction |
| P2-C | TGCGGCCGCTGCAGGTCGACGGATCCCCTTCTTCTTGATGGCAGCCTT | *BamHⅠ*, partial sequence of *Nb*eEF1A nt 1472-1452 |  |
| P3-N | CGCGGATCCTCAGAAGAAGAAATGAACGTT | *BamHⅠ*, partial sequence of *Nb*eEF1A nt 1451-1471 | TRV:*NbeEF1A* construction |
| P3-C | TCCCCCGGGAATTAAGTATCTTAAGCAACA | *SmaⅠ*, partial sequence of *Nb*eEF1A nt 1750-1730 |  |
| P4-N | GGGGACAAGTTTGTACAAAAAAGCAGGCTGCATGGGTAAAGAGAAATTTCAC | partial sequence of *Nb*eEF1A nt 154-174 | 35S:*Nb*eEF1A construction |
| P4-C | GGGGACCACTTTGTACAAGAAAGCTGGGTCTTTCTTCTTCTGAGCAGCCTT | partial sequence of *Nb*eEF1A nt 1494-1474 |  |
| P5-N | CCCTATAGTGAGTCGTATTATAAGTGTGTATACTATACATA | Partial sequence of CWMV RNA1 genomic nt 6730-6750 | + 3’UTR(RNA1) construction |
| P5-C | TGGGCCGGATAACCCTCCGGT | Partial sequence of CWMV RNA1 genomic nt 7147-7127 |  |
| P6-N | CCCTATAGTGAGTCGTATTATTGTGTATGTCCTTACTGACA | Partial sequence of CWMV RNA1 genomic nt 3174-3194 | + 3’UTR(RNA2) construction |
| P6-C | TGGGCCGGTTTACCCACCGGT | Partial sequence of CWMV RNA1 genomic nt 3569-3549 |  |
| P7-N | TAAGTGTGTATACTATACATA | Partial sequence of CWMV RNA1 genomic nt 6730-6750 | - 3’UTR construction |
| P7-C | TAATACGACTCACTATAGGGTGGGCCGGATAACCCTCCGGT | Partial sequence of CWMV RNA1 genomic nt 7147-7127 |  |
| P8-N | CCCTATAGTGAGTCGTATTAGTATTTCTTTCTCTCTACGTC | Partial sequence of CWMV RNA1 genomic nt 1-21 | + 5’UTR construction |
| P8-C | TTTGTTACAAACAAAGGATTT | Partial sequence of CWMV RNA1 genomic nt 101-81 |  |
| P9-N | GTATTTCTTTCTCTCTACGTC | Partial sequence of CWMV RNA1 genomic nt 1-21 | - 5’UTR construction |
| P9-C | TAATACGACTCACTATAGGGTTTGTTACAAACAAAGGATTT | Partial sequence of CWMV RNA1 genomic nt 101-81 |  |
| P10-N | CCCTATAGTGAGTCGTATTAAACAGCTCCAACTTTTAAATG | Partial sequence of CWMV RNA1 ORF nt 301-321 | + ORF^301-781^ construction |
| P10-C | TCATGATCTTTATTCTTTTTT | Partial sequence of CWMV RNA1 ORF nt 781-761 |  |
| P11-N | AACAGCTCCAACTTTTAAATG | Partial sequence of CWMV RNA1 ORF nt 301-321 | - ORF^301-781^ construction |
| P11-C | TAATACGACTCACTATAGGGTCATGATCTTTATTCTTTTTT | Partial sequence of CWMV RNA1 ORF nt 781-761 |  |
| P12-N | ACGAGTGTGTTGTCTCACCTT | Partial sequence of CWMV CP nt 121-142 | Detection the CWMV CP |
| P12-C | AGCATTAGATATAGCCAACGA | Partial sequence of CWMV CP nt 400-421 |  |
| P13-N | GAACGATAGCCGGTACCCGGGGTATTTCTTTCTCTCTACGTC | *Smal I*, partial sequence of CWMV RNA1 genomic nt 1-21 | CWMV ΔR1 construction |
| P13-C | TTTGCGGACTCTAGAGGATCCTACTACGTAACCAATTTACTT | *BamH I*, partial sequence of CWMV RNA1 genomic nt 6987-6967 |  |
| P14-N | GAACGATAGCCGGTACCCGGGGTATTTCAATCTGTACAAGTG | *Smal I*, partial sequence of CWMV RNA2 genomic nt 1-21 | CWMV ΔR2 construction |
| P14-C | TTTGCGGACTCTAGAGGATCCCGGGACGCCCAGTCGCTATTT | *BamH I*, partial sequence of CWMV RNA2 genomic nt 3406-3386 |  |
| P15-C | TTTGCGGACTCTAGAGGATCCTGGGTTACCCACCGGTTTGGG | *BamH I*, partial sequence of CWMV RNA2 genomic nt 3569-3544 | MCWM^Δ3561-3567^ construction |
| P16-C | TTTGCGGACTCTAGAGGATCCTGGGGGCCATTACCCACCGGT | *BamH I*, partial sequence of CWMV RNA2 genomic nt 3569-3549 | MCWMV^ugaacau^ construction |
| P17-C | TGGGGGCCATTACCCACCGGT | partial sequence of CWMV RNA2 genomic nt 3569-3549 | m3’UTR^ugaacau^ |
| P18-N | GGGGACAAGTTTGTACAAAAAAGCAGGCTGCATGCCGATCGATAGCAGTTCG | partial sequence of CWMV RNA1 genomic nt 102-122 | 35S:Met construction |
| P18-C | GGGGACCACTTTGTACAAGAAAGCTGGGTCCAAACCCCAAGCATATTTAGA | partial sequence of CWMV RNA1 genomic nt 1830-1810 |  |
| P19-N | GGGGACAAGTTTGTACAAAAAAGCAGGCTGCATGTCTAAATATGCTTGGGGTTTG | partial sequence of CWMV RNA1 genomic nt 1983-2003 | 35S:Hel construction |
| P19-C | GGGGACCACTTTGTACAAGAAAGCTGGGTCTTTGTCGAATTCAGGACACAA | partial sequence of CWMV RNA1 genomic nt 4151-4131 |  |
| P20-N | GGGGACAAGTTTGTACAAAAAAGCAGGCTGCATGTGTCCTGAATTCGACAAATGA | partial sequence of CWMV RNA1 genomic nt 4135-4155 | 35S:RdRp construction |
| P20-C | GGGGACCACTTTGTACAAGAAAGCTGGGTCCCATTCAAAGTTCCTATCTAC | partial sequence of CWMV RNA1 genomic nt 5675-5655 |  |
| P21-N | GGGGACAAGTTTGTACAAAAAAGCAGGCTGCATGGCGAAAGGTAAGAGGCAA | partial sequence of TuMV genomic nt 5903-6013 | 35S:VPg-Pro construction |
| P21-C | GGGGACCACTTTGTACAAGAAAGCTGGGTCTTGTGCGTAGACTGCCGTGCT | partial sequence of TuMV genomic nt 7207-7187 |  |
| P22-C | TACTACGTAACCAATTTACTT | partial sequence of CWMV RNA1 genomic nt 6987-6967 | 3’UTR-A (RNA1) |
| P23-N | CCCTATAGTGAGTCGTATTACAGTGCGTTAAACTGTACGAC | partial sequence of CWMV RNA1 genomic nt 7127-7147 | 3’UTR-B (RNA1) |
| P24-C | CGGGACGCCCAGTCGCTATTT | partial sequence of CWMV RNA2 genomic nt 3406-3386 | 3’UTR-A (RNA2) |
| P25-N | CCCTATAGTGAGTCGTATTATTGTGTATGTCCTTACTGACA | partial sequence of CWMV RNA2 genomic nt 3533-3569 | 3’UTR-B (RNA2) |
| P26-N | CCCTATAGTGAGTCGTATTAGTCCCGCAGTACGTTTAATAT | partial sequence of CWMV RNA2 genomic nt 3407-3438 | 3’UTR^Δ3419-3429^ |
| P27-C | TGGGTTACCCACCGGTTTGGG | partial sequence of CWMV RNA2 genomic nt 3569-3544 | 3’UTR^Δ3561-3567^ |
| P28-N | GTGACAGGCCCGAATCGAGCA | partial sequence of CWMV RNA1 genomic nt 6050-6070 | Detection the replication of CWMV RNA1 |
| P28-C | TTCCAAAGATGTCAAAGGTGA | partial sequence of CWMV RNA1 genomic nt 5765-5745 |  |
| P29-N | GAAGCGTTTACTCCGTGAGAA | partial sequence of CWMV RNA2 genomic nt 510-530 | Detection the replication of CWMV RNA2 |
| P29-C | CGCTGCGCTGGATAGGTTCAG | partial sequence of CWMV RNA2 genomic nt 220-200 |  |

**Note:** a, Underlined letters indicate restriction enzyme sites, b, Numbers correspond to target nucleotide positions; a reverse order of numbers indicates that the primer is complementary to the targeted sequences.
